# The challenge of preventing extinctions: Lessons from managing threatened land snails on Norfolk Island

**DOI:** 10.1371/journal.pone.0314300

**Published:** 2024-12-16

**Authors:** Isabel T. Hyman, Monique Van Sluys, Junn Kitt Foon, Nicholas A. Macgregor, Alexandra H. Anderson, Tara Patel, Tarryn Williams Clow, Melinda Wilson, Andrew Daly, Kerrie Bennison, Parnee Bonson, Simon Brown, Brendon Christian, Brett Finlayson, Nigel Greenup, Lilli-Unna King, Frank Köhler

**Affiliations:** 1 Australian Museum Research Institute, Sydney, Australia; 2 Invertebrates Australia, Osborne Park, Australia; 3 Taronga Conservation Society Australia, Mosman, Australia; 4 School of Science, Western Sydney University Hawkesbury Campus, Richmond, Australia; 5 Parks Australia, Canberra, Australia; 6 Durrell Institute of Conservation and Ecology (DICE), School of Anthropology and Conservation, University of Kent, Canterbury, United Kingdom; 7 Parks Australia, Norfolk Island National Park and Botanic Gardens, Norfolk Island, Australia; 8 Norfolk Island Regional Council, Norfolk Island, Australia; 9 Parks Australia, Christmas Island National Park, Christmas Island, Australia; 10 Parks Australia, West and North Marine and Island Parks, Western Australia, Australia; 11 Department of Infrastructure, Transport, Regional Development, Communications and the Arts, Canberra, Australia; Federal University of Juiz de Fora, BRAZIL

## Abstract

Norfolk Island, situated between Australia, New Zealand and New Caledonia, has a rich, narrowly endemic land snail fauna, which has suffered considerably from habitat loss and introduced predators. Eleven species (Stylommatophora, Microcystidae) are currently listed by the IUCN and/or Australia’s EPBC Act 1999 as Endangered, Critically Endangered or Extinct. Based on targeted surveys undertaken since 2020, we re-assess the threat status of these taxa. For three species assessed to be at imminent risk of extinction, we report on the implementation of *in-situ* and *ex-situ* conservation actions and assess their effectiveness after a three-year period. We document current distributions and abundances of these species and describe experimental conservation methods, such as increased predator control, the erection of predator-proof exclosures, and the establishment of an *ex-situ* breeding population. We found that the relative abundance of one subspecies, *Advena campbellii campbellii*, was strongly correlated with monthly rainfalls. Trials of predator-proof exclosures that retain adults but allow juveniles to disperse indicated that snails can be successfully secured from predation. Increased efforts in predator control led to the killing of more rodents and chickens; however, the impact on the snail population is unclear. The *ex-situ* breeding population had high birth rates initially followed by high adult mortality. Adjustments in husbandry conditions reduced stress levels leading to sustainable birth rates and increased survivorship with the result of rapid population growth. We determined that the ovoviviparous *A*. *campbellii campbellii* matures at the age of 3–4 months and has a lifespan of 10–12 months in captivity. We conclude that focused predation studies are needed to determine the impact of introduced predators. The use of exclosures requires further refinement especially regarding feeding schedules. *In-situ* breeding requires significant time for establishment but can be implemented successfully. We assess three endemic species as Extinct, four as Critically Endangered and two as Vulnerable.

## Introduction

**Fig 1:**
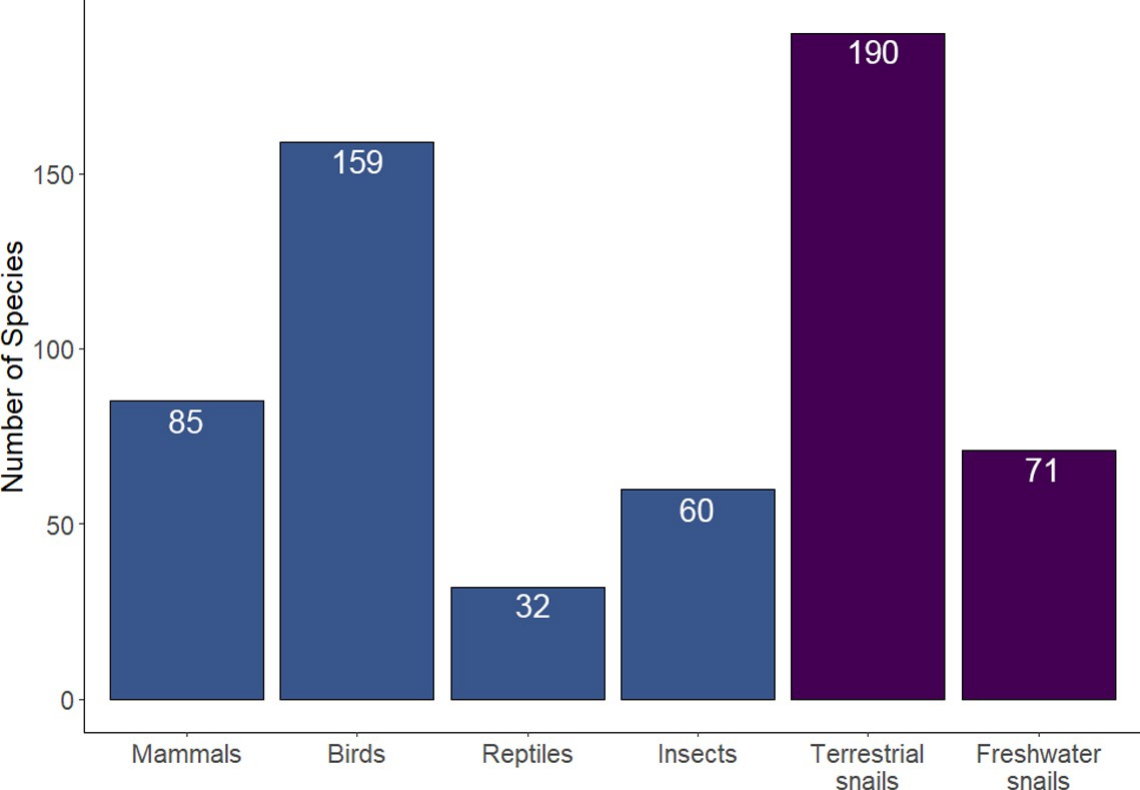
The number of species listed as Extinct on the IUCN Red List of threatened species across different animal groups [2].

Land snails have more recorded extinctions than birds, reptiles, mammals or insects [1, 2] (Fig 1). Their decline is most alarming throughout the islands of the Indo-Pacific, where it is driven primarily by introduced predators, particularly the rosy wolf snail (*Euglandina rosea*) and the New Guinea flatworm (*Platydemus manokwari*) [3]. Predation by rats (the black rat, *Rattus rattus*, the brown rat, *Rattus norvegicus*, and the Polynesian rat, *Rattus exulans*) is another common cause of land snail and other invertebrate declines and extinctions on oceanic islands [1, 4] as is anthropogenic habitat modification and destruction [5].

Snails on islands, like other island-dwelling fauna, are particularly susceptible to disturbances for several reasons. First, they have often evolved in the absence of certain groups of predators; for example, many islands have no native mammals nor carnivorous snails, and the endemic snails have therefore evolved no defenses against them. They may also be vulnerable to diseases or parasites brought in by introduced snail species. Second, the ranges of island species are restricted by island size rendering them particularly vulnerable to the effects of habitat destruction and modification. Third, many islands will increasingly be affected by climate change, including the effects of sea level changes, rising temperatures, and altered rainfall patterns, which affect the distribution of certain vegetation types with cascading impacts on animals [1, 5–9]. The spatial constriction on islands limits opportunities to escape these changes.

In March 2020, two of the authors (IH & FK) undertook the first targeted land snail survey in many years on Norfolk Island, one of Australia’s hotspots of land snail diversity in terms of the number of endemic species in a comparatively small area. The island is home to approximately 60 endemic species [10–13]. During this survey, we recorded 35 species, including *Advena campbellii* (drawn to our attention by Norfolk Island resident Mark Scott) and *A*. *suteri* (Stylommatophora: Microcystidae). Both species are listed as Critically Endangered under the Australian Environment Protection and Biodiversity Conservation (EPBC) Act (1999), and as Extinct (*A*. *campbellii*) or Endangered (*A*. *suteri*) on the IUCN Red List of Threatened Species. In 2020 they were each recorded in a single, very small area with fewer than 25 living specimens observed. This triggered concerns that both species may have reached a critical conservation status. In response to these findings, an alliance was formed by conservation managers and scientists from Parks Australia, Norfolk Island Regional Council, Taronga Conservation Society Australia and the Australian Museum Research Institute. This alliance has begun work to identify and implement appropriate interventions to bolster populations of the two species and any other species of concern on the Norfolk Island group against further decline. Herein we outline the results of the initial three years of conservation management, and present updated conservation assessments for the threatened land snails of Norfolk Island.

**Fig 2:**
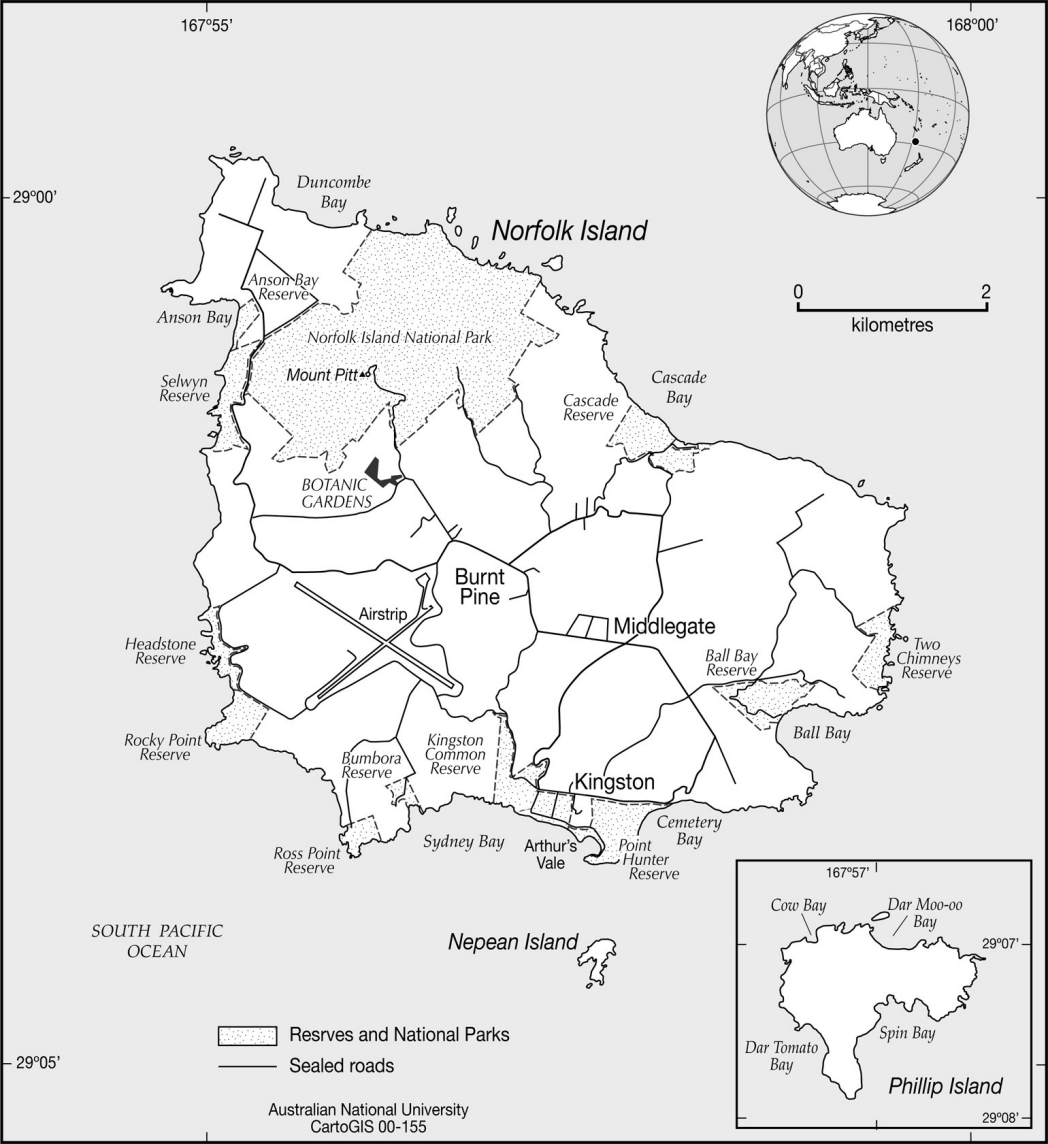
Map of the Norfolk Island group. Republished from CartoGIS Services, College of Asia and the Pacific, The Australian National University under a CC BY license, with permission from CartoGIS Services.

The Norfolk Island group consists of three islands situated in the South Pacific Ocean, approximately 1,500 km from the Australian mainland, 1,000 km from New Zealand and 750 km from New Caledonia (Fig 2). The largest two islands, Norfolk Island (35 km^2^) and Phillip Island (2 km^2^), are volcanic in origin and were formed between 2.3 and 3.1 million years ago. By contrast, the third island (Nepean Island, 0.04 km^2^) is calcareous and of more recent origin [14]. On the southern side of Norfolk Island, facing Nepean Island, there are also thick deposits of calcarenite [15].

The three islands have been impacted in different ways by human settlement. Norfolk Island was settled by Polynesians from the Kermadec Islands from the 13th to the 15th centuries [15]. British settlers arrived in 1788 and Norfolk Island was then occupied by convicts and free settlers until 1814. A second period of convict settlement occurred from 1825 to 1855. In 1856, the island was entrusted to settlers from Pitcairn Island [16]. During all periods of human settlement, extensive clearing of native subtropical rainforest occurred, creating the pastoral landscape that is seen across most of the island today. A plethora of introduced plant and animal species have become established on Norfolk Island, including the Polynesian rat (*Rattus exulans*), black rat (*R*. *rattus*), house mouse (*Mus musculus*), cat (*Felix catus*), chicken (*Gallus domesticus*), Argentine ant (*Linepithema humile*), cherry guava (*Psidium cattleyanum*) and African olive (*Olea europaea cuspidata*) [17], as well as three species of land Planaria, the Kontikia flatworm (*Kontikia ventrolineata*), the blue garden flatworm (*Caenoplana coerulea*) and the shovel-headed garden worm (*Bipalium kewense*) [pers. obs.,^18^].

Pigs, goats, rabbits and chickens were released on Phillip Island in the late 1700s to provide a reliable source of food for convicts and settlers [19]. These animals denuded the vegetation on Phillip Island to such an extent that by the early 1900s it appeared as a barren wasteland, with virtually no vegetation remaining and much soil eroded. The last feral rabbit was killed in 1988 and since then vegetation has begun to return, but the island is largely dominated by invasive species. Importantly, rats were never introduced to Phillip Island, nor was the island permanently populated by humans. So, despite the widespread devastation, there are species present on Phillip Island that are not seen on Norfolk Island, including an endemic centipede (*Cormocephalus coynei)*, two reptiles that are endemic to the Norfolk Island group and Lord Howe Island (the Lord Howe Island gecko, *Christinus guentheri*, and the Lord Howe Island skink, *Oligosoma lichenigera*), and many sea birds [17, 19].

Nepean Island was once vegetated with open forest dominated by around 200 Norfolk Island pines [20], but was cleared between 1790 and 1840 and by 1835 was described as ‘very sterile’ [21]. The vegetation has never recovered.

The earliest descriptions of the land snails of the Norfolk Island group [22] are based on samples collected during an expedition to Phillip Island in 1830. Over the next 80 years, there were additional species descriptions through the taxonomic literature mostly based on specimens traded by shell dealers [23–25]. The most significant contributions were enabled by the collections of Roy Bell, who moved to Norfolk Island in 1910 and collected comprehensively. Based on this material, revisions were carried out by Preston [9] and Iredale [10], bringing the total number of accepted species to about 70. Revisions by Smith [11] and Hyman *et al*. [12] have reduced that number somewhat, currently to 61 native species, 59 of these endemic. Norfolk Island’s land snail fauna is dominated by the ovoviviparous stylommatophoran family Microcystidae (27 species) [12]. Most of the island’s threatened snails belong to this family (including the focal taxa of this study, *Advena campbellii campbellii*, *A*. *suteri* and *F*. *imitatrix*). Partulidae and Achatinellidae, two different families of Pacific Island snails, also have an ovoviviparous reproductive strategy. Both groups have been devastated by predation from introduced species across many islands of the Pacific [4, 26, 27] and we hypothesize that comparatively lower reproductive rates in these ovoviviparous lineages contribute to their increased susceptibility.

Nineteen of the Norfolk Island endemic species were assessed for the IUCN Red List in 1996, resulting in five species being listed as Extinct (EX), four as Endangered (EN), eight as Vulnerable (VU), and two as Data Deficient (DD) (Table 1) [2]. Three of these and two additional species were listed as Critically Endangered (CR) under the Australian Government’s EPBC Act (1999) [28–32].

**Table 1:** Norfolk Island land snails listed by the IUCN and the EPBC Act (1999).

	IUCN			EPBC	
Species (current name)	listing	criteria	assessed as	listing	assessed as
*Duritropis albocarinata*	DD	-	*Omphalotropis albocarinata*		
*Telmosena suteri*	DD	-	*Omphalotropis suteri*		
*Norfolcioconcha iota*	VU	D2	*Norfolcioconch iota*		
*Norfolcioconcha norfolkensis*	VU	D2	*Norfolcioconch norfolkensis*		
*Penescosta mathewsi*	VU	D2			
*Penescosta sororcula*	VU	D2			
*Advena campbellii* *	EX		*Advena campbelli*	CR	
*Advena charon*	EN	B1+2c			treated as synonym of *A*. *campbellii*
*Advena grayi* *				CR	*Mathewsoconcha grayi*
*Advena phillipii* *				CR	*Mathewsoconcha phillipii*
*Advena suteri* *	EN	B1+2c	*Mathewsoconcha belli*	CR	*Mathewsoconcha suteri*
*Allenoconcha caloraphe*	VU	D2	*Iredaleoconcha caporaphe*		
*Allenoconcha quintalae* *	EX		*Nancibella quintalae*		
*Allenoconcha retinaculum*	VU	D2	*Buffetia retinaculum*		
*Fanulena amiculus* *	EN	B1+2c	*Dolapex amiculus*		
*Fanulena imitatrix* *	EN	B1+2c	*Lutilodix imitatrix*		
*Fanulena perrugosa* *	EX		*Panulena perrugosa*		
*Pittoconcha concinna*	VU	D2			
*Quintalia flosculus* *	EX				treated as subspecies of *Q*. *stoddartii*
*Quintalia stoddartii* *	EX			CR	
*Christianoconcha quintali*	VU	D2	*Christianoconcha quintalia*		

All listed species are endemic to Norfolk Island. Species with an asterisk (*) are reassessed in the current study. The name under which the species was assessed is given only if it differs to the currently accepted species name. Abbreviations: CR, critically endangered; DD, data deficient; EN, endangered; EX, extinct; VU, vulnerable.

In the current study we present updated species assessments of all eleven species listed as EN, CR or EX under either the IUCN Red List and/or the EPBC Act (1999), document preliminary results of the conservation actions taken to protect *A*. *campbellii*, *A*. *suteri* and *F*. *imitatrix*, three species deemed at highest risk, and make recommendations for future work. Specifically, we discuss the outcomes and lessons learnt for the following conservation research and management actions: (1) documentation of distribution; (2) documentation of body size and abundance; (3) *ex-situ* methods / husbandry program; (4) habitat enhancement and predator control; (5) predator exclusion; (6) monitoring; and (7) species conservation assessments.

## Methods

### Ethics statement

This study does not involve human subjects. The animal subjects that form the focus of our study are invertebrates (land snails) for which ethics approval is not required. Control of introduced vertebrate predators (rats and chickens) was carried out; however, introduced predator control to protect native species is a legal requirement of the staff of the Norfolk Island National Park and does not require ethics approval [17]. This study involves field work, under permits NINP 2020/R/01, 2020/12 and 2020/R/14 of the Norfolk Island National Park, and permits 11 of 2020, 34 of 2020, 28 of 2021 and 20 of 2022 of the Norfolk Island Regional Council. Field work was carried out on protected land under the permits listed above, and on a few occasions private land was accessed with permission from the landowners. Protected species were sampled under the permits listed above. Specimens of *A*. *campbellii campbellii* and *A*. *suteri* were collected for the husbandry programme under EPBC permit E2020-0182. Animal husbandry was undertaken at Taronga Conservation Society.

### Documenting distribution

**Fig 3:**
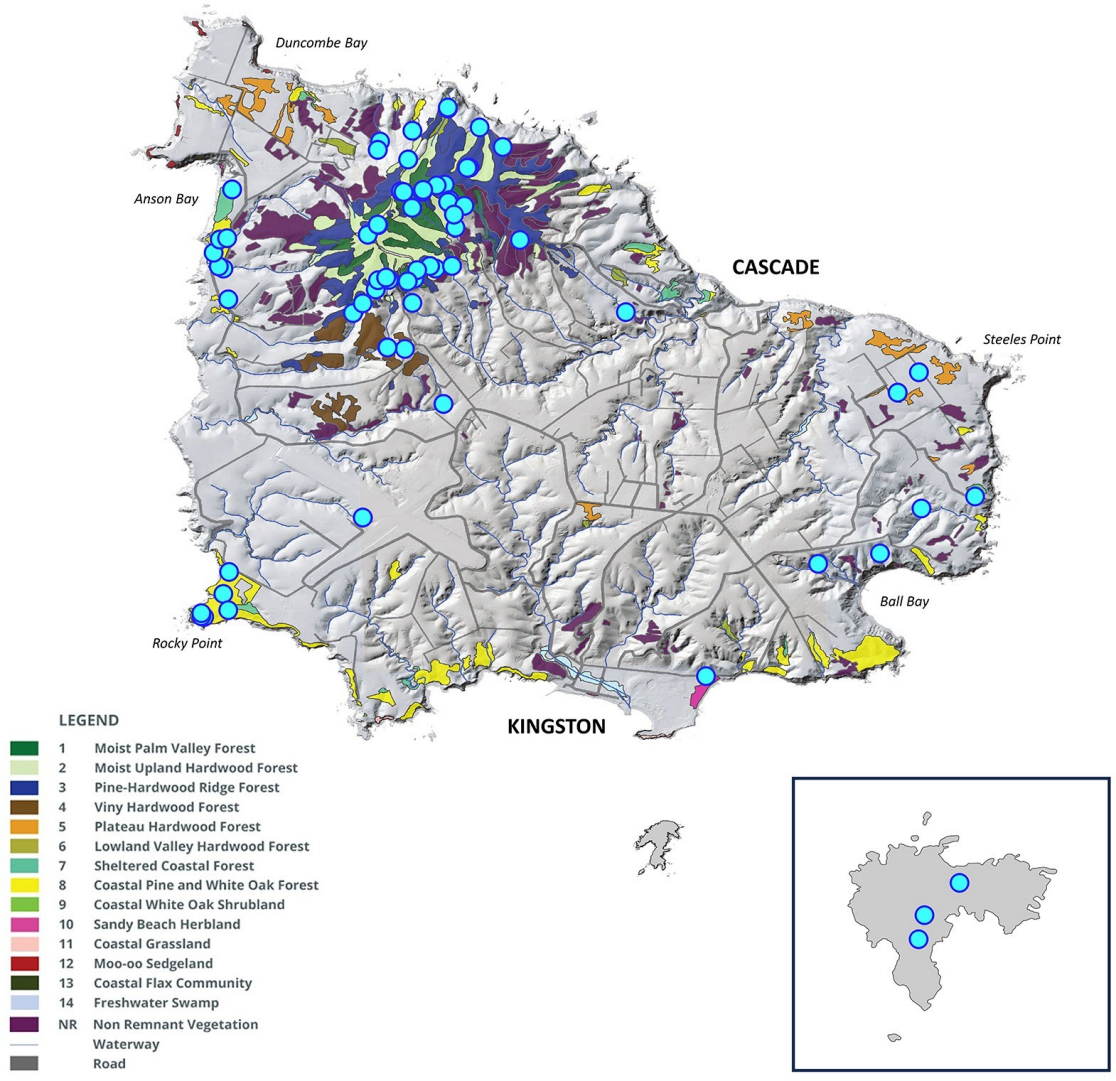
Map of surveyed areas. Areas of remnant native vegetation are marked in colour by vegetation type (see key) while cleared areas are shown in grey. The inset map shows Phillip Island. Map republished from [33] under a CC BY license, with permission from the Invasive Species Council, original copyright 2009.

Land snail surveys were undertaken on six field trips between March 2020 and November 2022. Areas targeted included the Norfolk Island National Park and Botanic Gardens, any of the island’s public reserves that still contain remnant patches of native vegetation, and some private properties with remnant native vegetation [33] (Fig 3). General collecting by hand, targeting ground-dwelling, rock-dwelling and arboreal species, was supplemented by collection and sorting of leaf litter. Initial searches were untimed, focusing on comprehensive coverage of different habitat types rather than quantitative assessments of abundance. This method prioritises sampling as broadly as possible to allow for rare species to be detected. All previously collected samples from the Australian Museum Malacology collection were also examined. Samples were divided into three categories according to their collection years: (1) recent records refer to live or freshly dead specimens collected from 2000 to present; (2) historical records refer to live or freshly dead specimens collected before 2000; (3) subfossil records refer to worn specimens that were not freshly dead at the time of collection, often collected from core samples at limestone sites and likely to predate modern human occupation. Identifications were made using the most up-to-date taxonomic literature [11, 13]. All threatened species could be readily identified based on shell parameters, including dimensions, colour, sculpture and ornamentation. Based on these data, distribution maps were created for each species.

### Estimation of abundance and body size

*Advena campbellii campbellii*, known only from three restricted sites in moist palm valleys in the Norfolk Island National Park, and *A*. *suteri*, known only from one site in open white oak and Norfolk Island pine forest in Hundred Acre Reserve, were assessed for abundance by conducting timed searches and counts of living snails at all their known locations on the first three trips of the project (March 2020, October 2020 and May 2021). In February, May and November 2022, we also measured the shell width of all specimens as an indicator of each specimen’s relative age. These surveys were not timed because of the additional time needed for the measuring. Shell width was used as a proxy for maturity as it can quickly and easily be measured in the field with minimal disturbance to snails and appears to be a good indicator of reproductive maturity [34]. Living individuals of *Fanulena imitatrix* were also counted on three occasions in Selwyn Reserve.

### Collection of living snails and husbandry program

In May 2021 we collected 16 living individuals each of *A*. *campbellii campbellii* and *A suteri* to develop husbandry programs and establish insurance populations at Taronga Zoo (Sydney, Australia) (EPBC permit number E2020-0182). In May 2022, we collected an additional 30 living specimens of *A*. *campbellii campbellii*. Snails were housed in perspex tanks in a humidity- and temperature-controlled environment with a natural day-night cycle very similar to that of Norfolk Island. Each tank houses snails of varying ages, with usually 5–10 adults and a variable number of juveniles and neonates (to a maximum of 30 snails in total). Births and deaths for each tank were recorded. Due to strict quarantine requirements between Norfolk Island and mainland Australia, the snails were kept in quarantine conditions with no natural vegetation being allowed. Snails were fed on a custom diet developed by Taronga Zoo, consisting primarily of oatmeal and nettle leaf, mixed into a paste and spread thinly on perspex plates [34]. Cardboard and paper hides were provided for use as shelter sites. Full details of the collection protocols and husbandry guidelines are provided by Daly *et al*. [34].

### Habitat enhancement and predator control

The three species identified as being at highest risk of extinction because of their very small population sizes are *A*. *campbellii campbellii* (Norfolk Island National Park), *A*. *suteri* (Hundred Acres Reserve) and *F*. *imitatrix* (Selwyn Reserve). At the locations where each of these species is found, habitat enhancement and/or predator control was implemented to protect and stabilise the wild populations. Around the sites of *A*. *campbellii campbellii* and *A*. *suteri*, rodent baiting and trapping and chicken culling was increased. The methods used included the deployment and regular baiting of GoodNature traps (model A24 Rat and Mouse Trap) for rodent control (four at the main *A*. *campbellii campbellii* site and three at the *A*. *suteri* site) and weekly chicken culling through shooting between November 2021 and October 2022.

In addition, in the habitats of *A*. *suteri* and *F*. *imitatrix*, both of which shelter under logs (pers. obs.), woody debris was added in the form of piles of small logs of Norfolk Island pine (*Araucaria heterophylla*). This was done in Hundred Acres Reserve in October 2021 (for *A*. *suteri*) and in Selwyn Reserve in mid-2022 (for *F*. *imitatrix*). The presence of coarse woody debris has been demonstrated to correlate with increased land snail abundance and diversity [35–38].

### Predator-proof exclosures

For *A*. *campbellii campbellii* and *A*. *suteri* we trialed cages designed to exclude rodents and chickens (exclosures) as a possible conservation management tool. Exclosures had wooden frames covered with 5 mm metal mesh, to protect breeding adults while allowing neonates to disperse (design based on [39]). Exclosures, 90 cm long x 90 cm wide x 30 cm high, were deployed at two sites: the main site of *A*. *campbellii campbellii* in the Norfolk Island National Park, and the only known site of *A*. *suteri* in Hundred Acre Reserve.

Experiments were carried out in two phases. In phase 1, two exclosures and two controls were prepared for each of the two sites. Controls consisted of the same wooden frame with mesh on the top but not on the sides. Controls and exclosures were deployed 10–20 m apart. Ten snails, individually marked in a unique fashion with water-based enamel paint, were placed in each exclosure / control, along with suitable litter / logs / palm fronds to use for shelter and food. Exclosure placement was determined by searching for a suitably flat site that was already inhabited by living snails of the target species, which was taken as an indication that the microhabitat was suitable. The exclosures were checked weekly for 6 weeks and fresh leaf litter was provided on each occasion. Any neonates present were recorded at each check. This was recorded as evidence of breeding but was not intended to be a quantitative measure of reproductive output, since neonates are small enough to move through the mesh and leave the exclosure.

In phase 2, the controls were ceased for *A*. *suteri*, because of the difficulty in searching for snails without damaging the surrounding environment. The marking method was changed from enamel paint to a more durable nail polish which was re-applied regularly as required. Finally, the snails were checked and their leaf litter replenished at intervals of 2–4 weeks instead of weekly, and the shell width of all snails was measured at the start of the trial period and again for any snails that died. Phase 2 was implemented for nine weeks, and then continued for *A*. *campbellii campbellii* until all snails had died, which was a further 15 weeks.

### Monitoring

Relative abundance of the main population of *Advena campbellii campbellii* was monitored by surveys carried out every three months. Timed searches of 40 person-minutes per point were conducted around three fixed points nine times between November 2021 and November 2023. The points were at least 30 m from each other to minimise pseudoreplication, based on movement studies of land snails larger than *A*. *campbellii campbellii* showing maximum range or displacement to be 25–32 m over 11–16 days [40, 41]. Live snails were counted and categorized as juvenile (shell width below 15 mm) or adult (shell width above 15 mm) [34]. Empty shells were counted and categorized as either fresh (with intact periostracum) or old (worn periostracum). We also counted how many shells showed signs of predation, and whether these shells were fresh or old. All shells were removed from the survey area so they would not be counted in subsequent surveys. We compared the number of live adults, preyed-on shells and total shells found for all six sites across the study period. To assess the proportion of predated individuals relative to the population, we compared the ratio of predated shells to the total number of shells and analysed this using single-factor ANOVA in XLStatistics (ver. 5.76, Rodney Carr, Deakin University, see http://learnline.cdu.edu.au/lecturers/kclark/excel/xlss5.pdf). To test for a possible correlation between snail abundance and rainfall, we used XLStatistics to calculate the correlation coefficient between the number of live snails and monthly rainfall figures for Norfolk Island from the Bureau of Meteorology [42].

### Conservation assessments

We reviewed the conservation assessments of all species previously assessed as EN, CR or EX following IUCN Red List guidelines [43]. We used GeoCAT (available online at http://geocat.kew.org/) to calculate the extent of occurrence (EOO) and area of occupancy (AOO) for each species.

The extent of occurrence (EOO) of each species is the area contained within a convex hull polygon around all known occurrence records. The area of occupancy (AOO) is the area contained within 2x2 km squares around each occurrence record. Before calculation of EOO and AOO, records were first checked for taxonomic and spatial accuracy and subfossil records were excluded. Calculations were made based on both recent data (collected in or after 2000) and historical data (specimens collected before 2000).

## Results

### Documenting distribution

**Fig 4:**
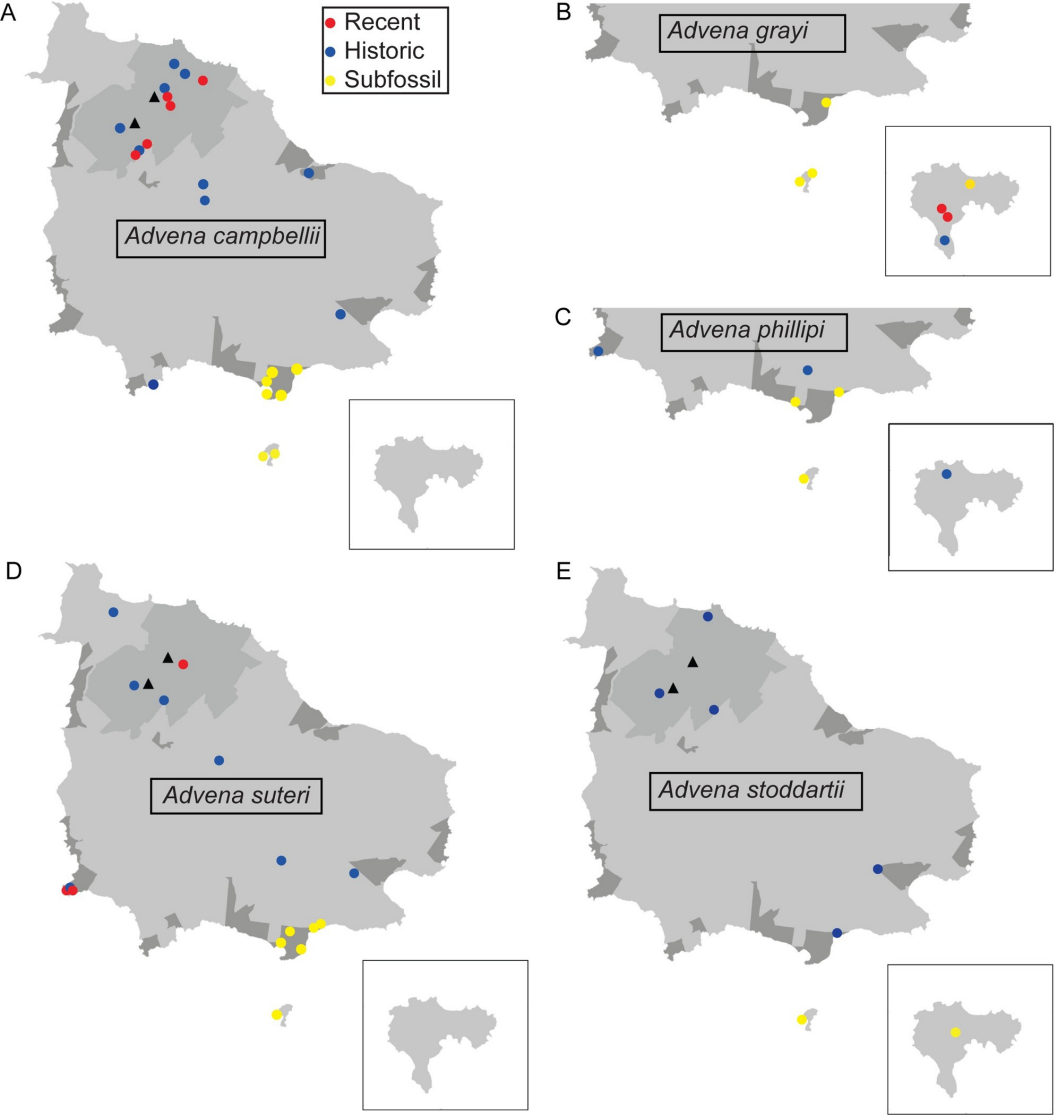
Distribution maps for *Advena* species. A. *Advena campbellii*. B. *Advena grayi*. C. *Advena phillipii*. D. *Advena suteri*. E. *Quintalia stoddartii*. The Norfolk Island National Park is shown in mid-grey and the Reserves in darker grey. Black triangles show Mt Pitt (lower left) and Mt Bates (upper right). The inset map shows Phillip Island. Recent records refer to live or freshly dead specimens collected from 2000 to the present. Historical records refer to live or freshly dead specimens collected before 2000. Subfossil records refer to worn specimens that are not freshly dead, often collected from core samples at limestone sites.

**Fig 5:**
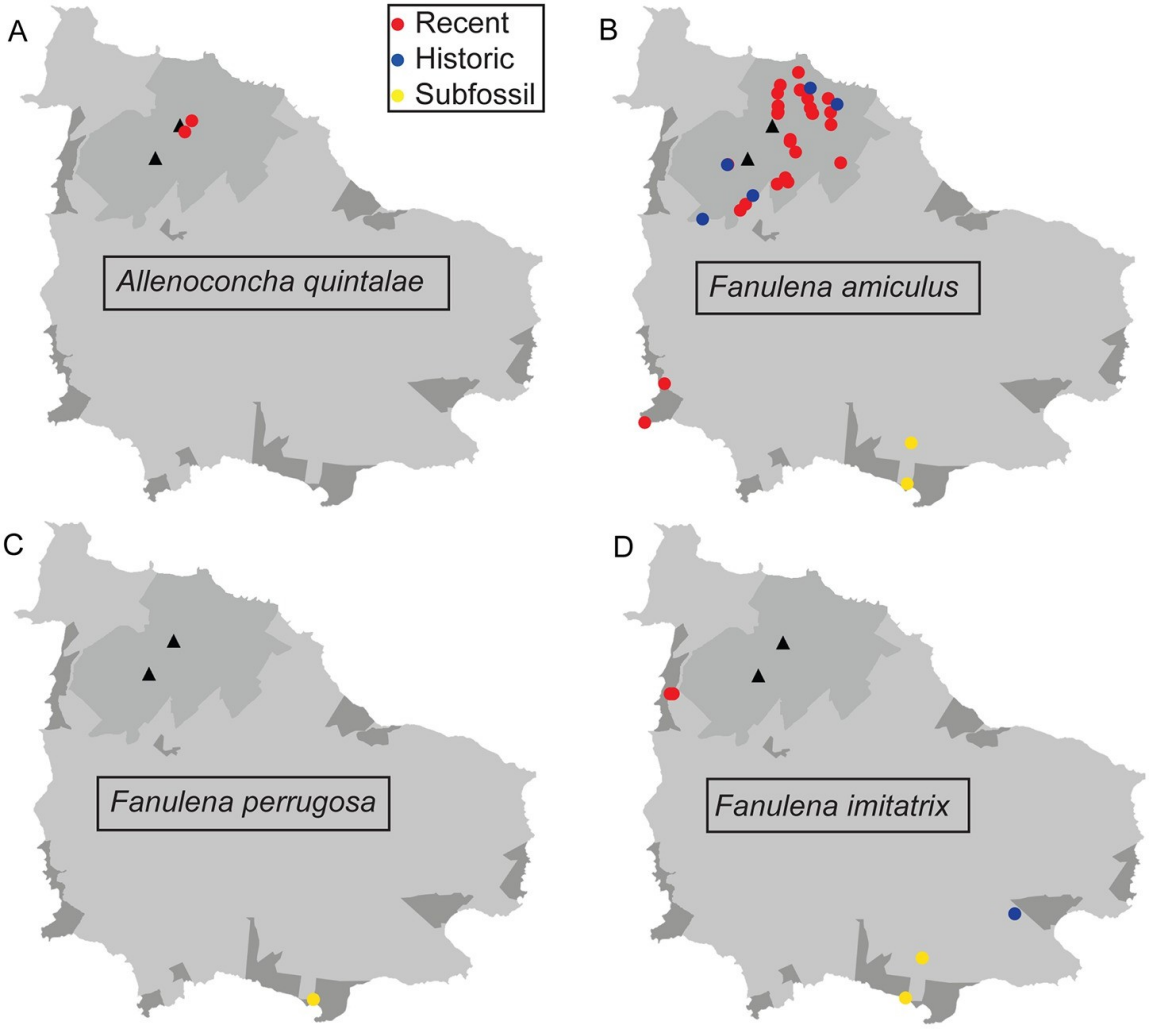
Distribution maps for *Allenoconcha* and *Fanulena* species. A. *Allenoconcha quintalae*. B. *Fanulena amiculus*. C. *Fanulena imitatrix*. D. *Fanulena perrugosa*. Details as for Fig 4.

Distribution maps of all species being assessed are shown in Figs 4 and 5. Of the nine species, six were recorded alive during our surveys (*A*. *campbellii*, *A*. *grayi*, *A*. *suteri*, *A*. *quintalae*, *F*. *amiculus* and *F*. *imitatrix*); the most recent verifiable collection records for the remaining three are from before 1945 (*A*. *phillipi*, *A*. *stoddartii* and *F*. *perrugosa*). Further distribution data are provided in S1 Appendix.

### Estimating abundance and size

**Fig 6:**
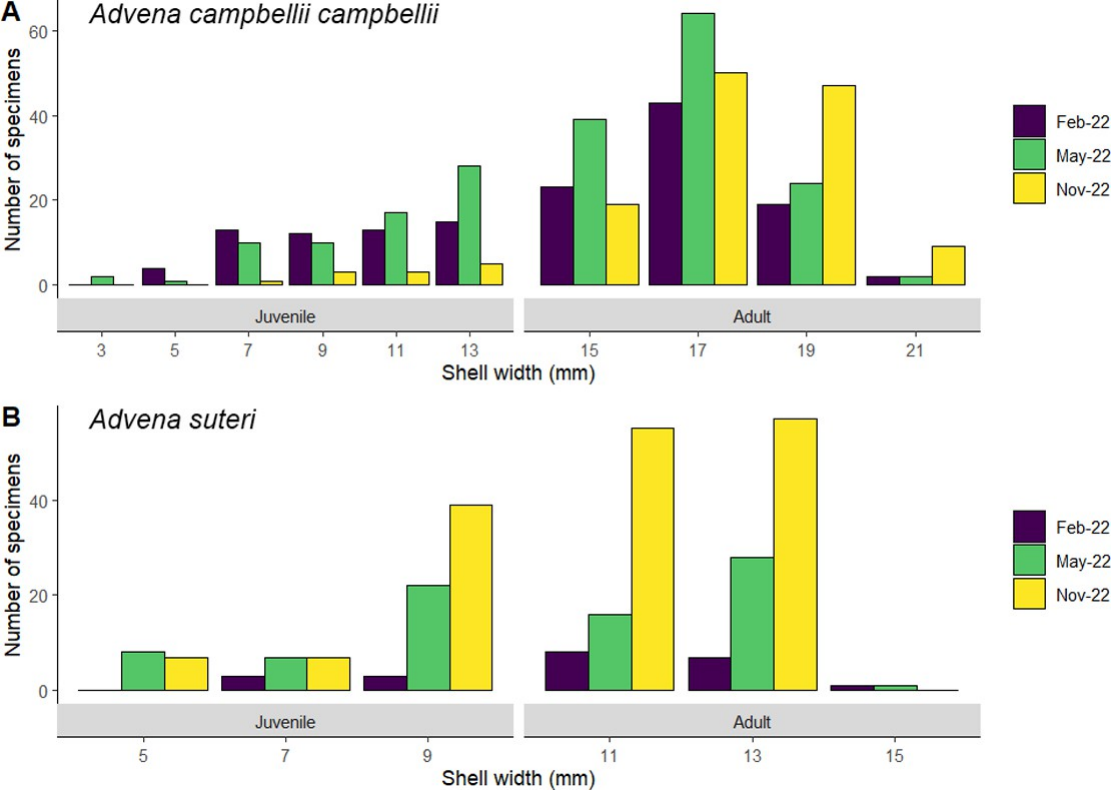
Abundance and shell width for populations of *Advena campbellii campbellii* (A) and *Advena suteri* (B) in February, May and November 2022.

Specimen counts of *A*. *campbellii campbellii*, *A*. *suteri* and *F*. *imitatrix* are shown in Table 2 and Fig 6. We observed higher numbers of both *A*. *campbellii campbellii* and *A*. *suteri* at the end of the study period (November 2022) than we did at the start of the study period (March 2020).

**Table 2:** Results of counts of *Advena campbellii campbellii*, *A*. *suteri* and *Fanulena imitatrix* in Norfolk Island, Australia.

	*Advena campbellii campbellii*				*Advena suteri*			*Fanulena imitatrix*
	sites	living snails	person-hours	snails / hour	living snails	person-hours	snails / hour	living snails
Mar-20	1	21	1.5	14	2	4.5	0.4	-
Oct-20	1	41	1.5	27	3	3	1	-
May-21	1	96	1.5	64	71	20	3.6	11
Feb-22	2	144	N/A	N/A	22	N/A	N/A	3
May-22	3	197	N/A	N/A	82	N/A	N/A	-
Nov-22	3	137	N/A	N/A	165	N/A	N/A	25

Shell width in *A*. *campbellii campbellii* (Fig 6A) ranged from 3 mm to 21 mm and the most abundant size category was 15–17 mm at all times of year. There were proportionally fewer juveniles in November 2022 (9%, compared to 35–40% in February and May), but the difference was not statistically significant.

In *Advena suteri* (Fig 6B), the most common size class was 9–11 mm in February 2022 and 11–13 mm in May and November 2022, and the maximum size was 15 mm. The proportion of juveniles present ranged from 27% (February) to 45% (May).

### Collection of living snails and husbandry program

At the outset of the husbandry program, the only life history information known about *A*. *campbellii campbellii* and *A*. *suteri* was that both species were ovoviviparous. This was confirmed when birthing of neonates was observed immediately after the first collection of snails in May 2021 (over the first 20 weeks, an average of 5 neonates per week in *A*. *campbellii campbellii* and an average of 5.6 neonates per week in *A*. *suteri*). However, after around 6 weeks we began to see mortality of adult founders. The observed decline was considered to result from unsuitable husbandry conditions, and a series of adjustments were implemented in diet, humidity, tank cleaning methods and other factors until the situation stabilized.

**Fig 7:**
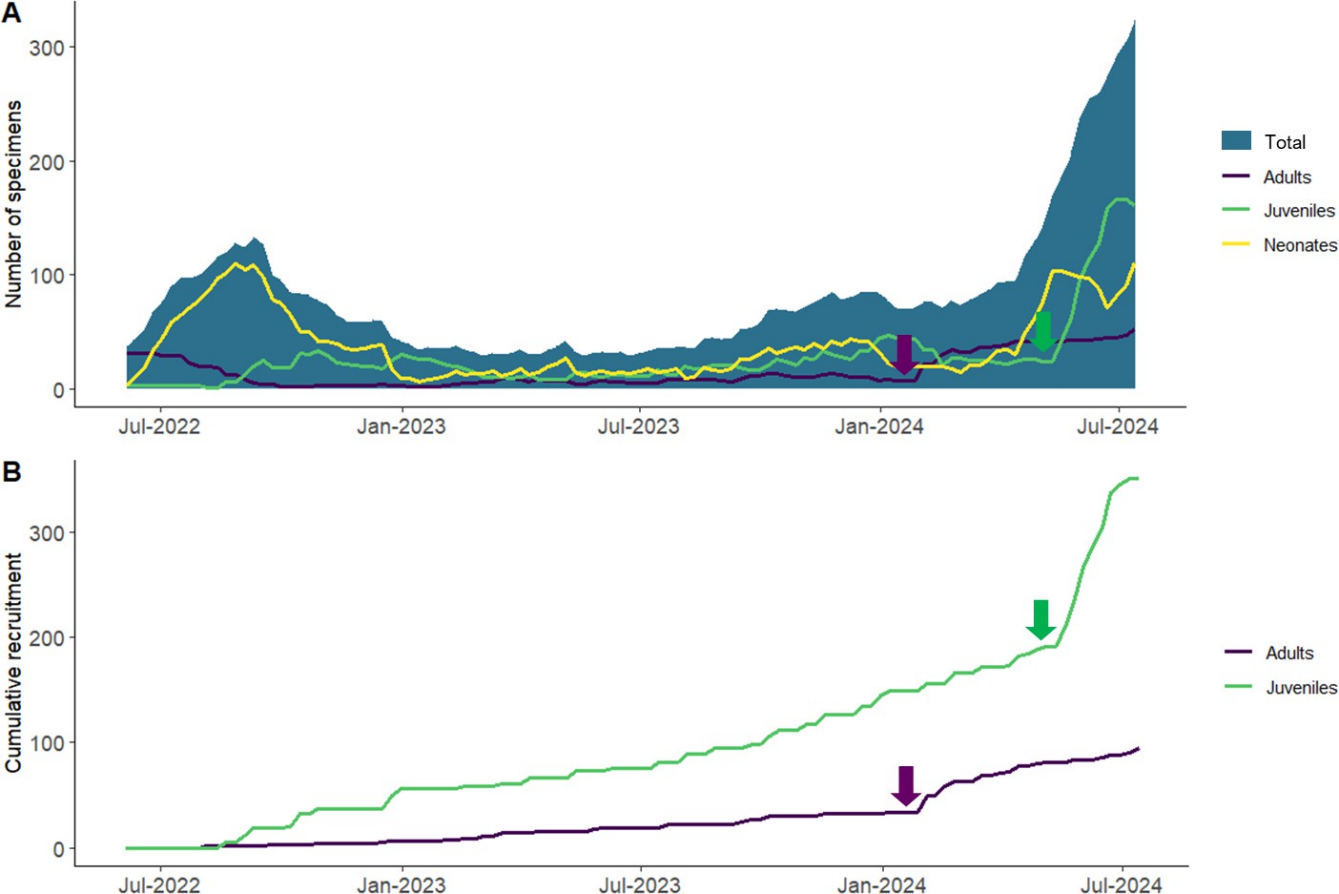
Snail numbers in the husbandry program from June 2022 to March 2024. A. Total adult count. B. Cumulative recruitment. In both A and B, the arrow on the left (in purple) shows the date from which a more hands-off approach to husbandry was implemented; the arrow on the right (in green) shows the date from which food was provided high on the walls of the tanks for neonates.

The factors which resulted in improvement in condition and increased survival of the snails included: (1) presentation of food. Early in the husbandry program, several adult *A*. *campbellii campbellii* and one *A*. *suteri* experienced everted mouthparts and died soon thereafter. The primary cause appeared to be the hard perspex surface upon which food was presented. When food was ground more finely, spread more thinly and spread upon a soft surface (damp paper towel), the problem resolved. In January 2024, after noting that neonates tend to climb and often aestivate far from the food plates, we started to target-feed them by adding smears of food paste to the fronds upon which they sit. In April 2024, we replaced this step with extra feeding sites high on the walls of the tanks, which are used primarily by neonates and young juveniles. Since this time we have seen a significant increase in neonate recruitment (Fig 7). (2) Humidity and brightness. Initially the tanks were very moist and the lights were on full brightness during the day, with no access to natural light. Current conditions include 70–90% humidity, and a natural day-light cycle and improved access to shaded areas, which appears to have been a factor in reducing stress. (3) Shelter sites. We replaced the paper hides with natural palm fronds, frozen and then microwaved to reduce risk of pathogen transmission, as another measure to reduce stress. (4) Tank cleaning. In the early days of the program, tank cleans (using sodium hypochlorite and detergent) often resulted in high mortality. The current regime (detailed in full by Daly et al. [34]) uses only hot water to clean tanks and minimises disturbance and handling of snails as much as possible. (5) Population structure and density. Initially neonates were removed from adults and placed in a separate tank. However, neonate mortality was high under this protocol, but decreased when neonates were retained in a tank with older snails. Observations indicated that neonates were more active in the presence of juvenile and adult snails. A higher tank density also appeared to be more successful. (6) Handling. While our approach was always as hands-off as possible, with minimal handling of snails, over the first 2.5 years of the program we still checked all palm fronds every day to determine where snails were positioned and check for unwell or dead snails. In February 2024, we implemented a more hands-off approach where daily observations were made without opening the tanks, and fronds were only comprehensively checked during the twice weekly feeding sessions. Since this change we have seen an increase in total adult count (Fig 7).

Despite these changes, which were implemented for both species, the population size of *A*. *suteri* did not recover and all snails died. We carried on with the captive population of *A*. *campbellii campbellii*, continuing to implement incremental changes to optimize the husbandry conditions as outlined above.

Our data indicate that in captivity, the ovoviviparous *A*. *campbellii campbellii* is reproductively mature from around 3–4 months of age. Mating has not been observed but, from maturity, most adults produce single neonates at regular intervals. In the early months of the program, the birth intervals averaged approximately 7–10 days [34]. However, between June 2022 and October 2023, longer average birth intervals of 16.1 days were observed. Short birth intervals have been attributed to high stress in other husbandry programs (D. Sischo, pers. comm.). Neonates born after a shorter birth interval were smaller, with thinner, less well-developed shells [34]. Average lifespan in captivity is 10–12 months, with a single adult living for 13 months [34]. The captive population had reached the seventh generation born in captivity by March 2024.

Currently, breeding is consistent, with birth occurring in all tanks and birth intervals ranging between 13.6 and 20.5 days across seven tanks during the period June 2022 to October 2023. Survivorship of young was around 9% for the period from June 2022 to June 2023, and around 33% for the period from June 2023 to March 2024 (Table 3). The captive population had increased to about 100 individuals by March 2024 (Fig 7) and to more than 400 in August 2024.

**Table 3:** Births and survivorship of *A*. *campbellii campbellii* in the husbandry program at Taronga Zoo, NSW, Australia.

	Total births	Neonate to juvenile	Juvenile to adult	Neonate to adult
June 2022 to May 2023	200	73 (36.5%)	19 (26%)	9.5%
June 2023 to March 2024	154	99 (64.3%)	51 (51.5%)	33.1%
**TOTAL**	**354**	**172 (48.6%)**	**70 (40.7%)**	**19.8%**

### Habitat modification and predator control

Piles of woody debris added to Hundred Acres Reserve in October 2021 and to Selwyn Reserve in mid-2022 were found in subsequent surveys to be used as shelter sites by moderately large numbers of *A*. *suteri* and *F*. *imitatrix*.

Over 100 chickens were culled between November 2021 and June 2022 across the *A*. *campbellii campbellii* and *A*. *suteri* sites, with the majority (97 chickens) being culled at Hundred Acres Reserve, the location of *A*. *suteri*. No chickens were observed at either site from June to October 2022, so chicken control measures were ceased at that point. It was noted that chickens appeared to learn to avoid staff members carrying a firearm. While this is merely observation and not supported by documented evidence, we mention it because this behaviour might have affected the number of observed chickens towards the end of the study period.

Rodent control measures resulted in 21 rats being killed at the *A*. *campbellii campbellii* site, but there was no uptake of bait and no kills at the *A*. *suteri* site.

### Predator-proof exclosures

#### Phase 1

At both sites, the snails in the control exclosures (without walls) dispersed and numbers gradually diminished. Our records of the rate of dispersal were hampered by problems with paint markings coming off, so results in weeks 3–4 were discarded and at four weeks a fresh set of ten control snails was marked for each control exclosure. Over both two-week periods, the ten *A*. *campbellii campbellii* in each control exclosure reduced to 0–1 specimens, while the ten *A*. *suteri* in each exclosure reduced to 4–5 specimens. Within the test exclosures, only one exclosure became insecure and had snails escape (*A*. *suteri*); this was rectified and no more snails escaped thereafter. Neonates were observed in three of the four exclosures across the six-week trial period, indicating that the enclosed snails were successfully breeding (see Table 4). By the end of the trial period, there were dead snails in each of the *A*. *campbellii campbellii* exclosures but none in the *A*. *suteri* exclosures (see Table 4).

**Table 4:** Results of the exclosure trial.

	*Advena campbellii campbellii*		*Advena suteri*	
	Excl. 1	Excl. 2	Excl. 1	Excl. 2
PHASE 1				
Maximum number of neonates	0	4	3	3
Number of dead snails	2	3	0	0
PHASE 2				
Maximum number of neonates	2	2	2	2
Number of dead snails	4	3	3	3
Shell width of dead snails (mm)	12.6–17.0	14.9–18.2	9.8–10.6	11.5–11.6
Average shell width of dead snails (mm)	14.7	16.7	10.1	11.6

The maximum number of neonates and number of dead snails in each exclosure for both phase 1 and phase 2 of the trials, Norfolk Island, Australia. Each exclosure began with a total of ten adult snails. Data for control exclosures are not shown. Maximum recorded adult size in the wild is 21 mm for *A*. *campbellii campbellii* and 15 mm for *A*. *suteri*.

#### Phase 2

The snails in the control exclosures (continued for *A*. *campbellii campbellii* only) dispersed away more slowly than they did in phase 1, with some snails being found in the control area after two and five weeks, respectively, but none by the final check after nine weeks. There was a slightly higher mortality rate in the test exclosures in phase 2: on average 35% in *A*. *campbellii campbellii* and 30% in *A*. *suteri*, compared to 25% and 0% respectively in phase 1 (see Table 4). A maximum of two neonates at any one time was observed in phase 2.

We continued phase 2 with *A*. *campbellii campbellii* until all snails were dead, an additional 15 weeks later. Thus, the maximum length of survival in an exclosure was 24 weeks. The average shell width at death in phase 2 including the extended period was 15.0 mm.

### Monitoring

**Fig 8:**
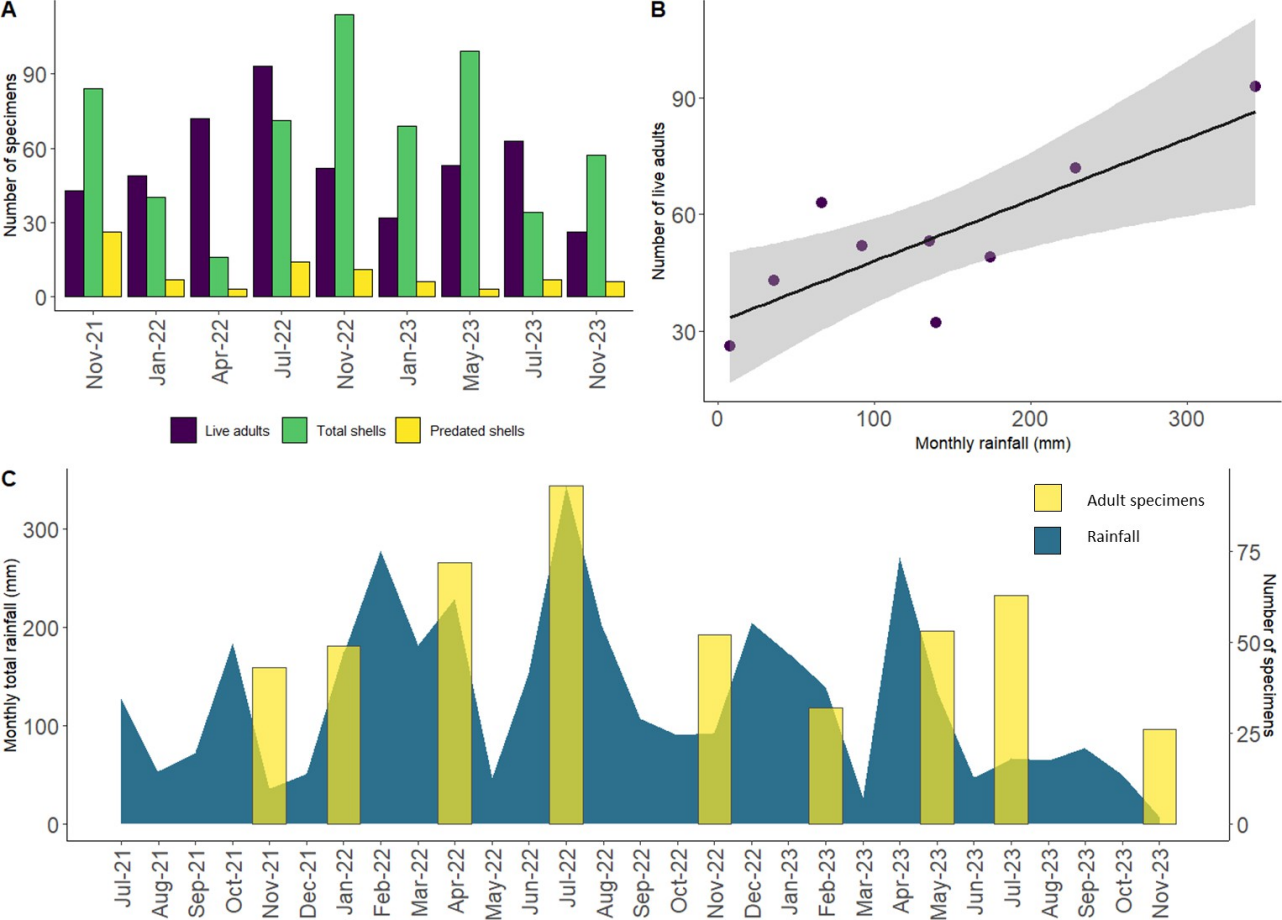
Results of regular monitoring of the main sub-population of *Advena campbellii campbellii* in the Norfolk Island National Park. A. Monitoring results. B. Number of live adult snails plotted against monthly average rainfall. C. Number of live adult snails (yellow bars) superimposed on monthly rainfall totals for Norfolk Island [42].

Monitoring around the main site of *A*. *campbellii campbellii* in the Norfolk Island National Park (Fig 8 and Table 5) showed that adult specimen counts were generally higher from April to July and lower in January and November, correlating strongly with monthly rainfall (correlation coefficient 0.80; Fig 8B and 8C). The number of freshly dead shells found peaked concurrently with a drop in live specimen count in November 2022, January 2023 and November 2023, suggesting increased mortality (rather than lower detectability). However, similar peaks in shell count occurred in July 2022 and May 2023 with no concurrent drop in live specimen count. Preyed-on shells were highest in the first survey in November 2021 (26 shells), with a second peak in July 2022, coinciding with the highest peak of live snails. When viewed as a ratio against the number of living adults, the proportion of predated shells was significantly higher in the first survey than in all the other surveys (p < 0.0005).

**Table 5:** Results of monitoring around the main site of *A*. *campbellii campbellii* in the Norfolk Island National Park.

Date	Live adults	Live juveniles	Total empty shells (fresh, old)	Preyed-on shells (fresh, old)	Preyed-on shells / total shells	Monthly total rainfall (mm)
Nov-21	43	0	84 (34, 50)	26	0.31	36.2
Jan-22	49	9	40 (23, 17)	7 (0, 7)	0.18	174.2
Apr-22	72	0	16 (14, 2)	3 (3, 0)	0.19	228.8
Jul-22	93	[unknown]	71 (29, 42)	14 (11, 3)	0.20	343.8
Nov-22	52	0	114 (31, 83)	11 (2, 9)	0.10	92.4
Jan-23	32	3	69 (24, 45)	6 (0, 6)	0.09	139.6
May-23	53	2	99 (37, 62)	3 (0, 3)	0.03	135
Jul-23	63	7	34 (24, 10)	7 (3, 4)	0.21	66.4
Nov-23	26	6	57 (16, 41)	6 (3, 3)	0.11	7.8

Total monthly rainfall figures are for the whole of Norfolk Island and were sourced from the Bureau of Meteorology [42].

### Species conservation assessments

The IUCN Red List currently has five Norfolk Island land snail species listed as EX, four as EN, eight as VU and two as DD, while the EPBC Act (1999) lists five species as CR. We assessed the conservation status of all nine species listed as EN, CR or EX by either the IUCN Red List or the EPBC Act (1999). Some inconsistencies were identified in current listings, with some species being listed under different names and with different statuses by the two bodies, several spelling errors in the IUCN listings, and taxonomic updates needed in both. Our assessments following IUCN criteria have resulted in three species recommended for listing as EX, four as CR and two as VU (Table 6). Full species assessments are available in S1 Appendix.

**Table 6:** Norfolk Island land snails assessed in the current study, showing their IUCN and EPBC listings and their updated status.

		IUCN		EPBC	Updated assessment		
Family	Species	listing	criteria	listing	listing	criteria	notes
Microcystidae	*Advena campbellii campbellii*	EX		CR	CR	B1+2ab	single location
Microcystidae	*Advena grayi*	-		CR	VU	D2	single location
Microcystidae	*Advena phillipii*	-		CR	EX		last seen 1830
Microcystidae	*Advena suteri*	EN	B1+2c	CR	CR	B1+2abc	single location
Microcystidae	*Advena stoddartii*	EX		CR	EX		last seen pre 1945
Microcystidae	*Allenoconcha quintalae*	EX		-	CR	B1+2ab	single location
Microcystidae	*Fanulena amiculus*	EN	B1+2c	-	VU	D2	Fewer than ten locations
Microcystidae	*Fanulena imitatrix*	EN	B1+2c	-	CR	B1+2ab	single location
Microcystidae	*Fanulena perrugosa*	EX		-	EX		known only from subfossil shells

## Discussion

### Insights from three years of surveys

Our observations of Norfolk Island’s snail fauna provide a series of snapshots in time, giving some insights into changes in species abundance and distribution. There are many possible factors that may have contributed to these changes. We note that abundance data predating our study have no documentation of search effort and therefore are not directly comparable with our new data, so we can reflect only upon changes in distribution.

Our observations indicate that for all species assessed there has been a decrease in EOO and AOO from historical to recent records. This reduction has predominantly occurred in the more distant past. During the past 10 years (the threshold for assessing population size reduction following IUCN guidelines [43]), however, no significant change in EOO or AOO has occurred. The most likely causes of past decreases include extensive land clearing, habitat degradation, introduced pests, and possibly climate change. To date, no studies have been conducted to understand the link between introduced weeds, habitat degradation and the decline of *Advena campbellii campbellii*. The specific environmental requirements for this species to persist, however, appear to be linked to intact native palm valleys and adjacent hardwood forest, suggesting that land clearing and the spread of invasive weeds pose a significant threat [44, 45].

Within the period of our surveys, we saw an initial increase in the abundance of *Advena campbellii campbellii* and *A*. *suteri* between March 2020 and May 2021. One key factor that may have impacted the species abundance is two years of drought preceding our first survey, with January 2020 being the driest month on record for Norfolk Island [42]. Another factor affecting abundance data for *A*. *campbellii campbellii* is the discovery of additional sub-populations by staff from the Norfolk Island National Park, which has been a positive outcome of increasing survey effort and knowledge of the species. As well as an overall increase in abundance over the course of our study period, we also observed the boundaries of the sites changing in both species over the three-year period. It is likely that distribution will fluctuate over a small scale with changing weather patterns, increasing in suitable weather and decreasing in extended dry periods. We discuss potential seasonality below under ‘Monitoring’.

Norfolk Island is experiencing increasingly drier weather and more extreme rain events as a result of climate change [46]. As well as directly affecting weather conditions, another outcome of climate change is an increase in the frequency and intensity of El Ninõ events, which cause reduced rainfall and hotter temperatures in eastern Australia [47, 48]. The potential impact of such conditions on species that are already severely limited in their extent and are experiencing predation pressure from introduced species cannot be understated.

During our surveys we have found living individuals of two species listed as Extinct by the IUCN Red List (*Advena campbellii campbellii* and *Allenoconcha quintalae*) and have also found living specimens of *Advena grayi* for the first time in 40 years. Living specimens of several other species never or only rarely recorded live in the past were also encountered (including *Fanulena imitatrix*, *Pittoconcha concinna* and *Allenoconcha retinaculum*). There has been an overall improvement in conservation listings of those species assessed, with the number of species listed as EX or CR decreasing and the number listed as VU increasing.

These findings demonstrate that intensive on-ground surveys to verify the extent of living sub-populations are crucial for improving the accuracy of conservation assessments of island land snails.

### Conservation management strategies

We have tested several conservation management strategies over the past three years, including the development of a husbandry program, the use of predator-proof exclosures, habitat enhancement, and predator control. Here we discuss the success and challenges of each of these strategies, and their broader applicability.

#### Husbandry program

The initial husbandry conditions we implemented were based on those developed for partulid tree snails [49]; however, it became evident that adjustments were necessary to account for the differences between the taxa. We had greater success when the food was presented on a soft rather than a hard surface, and when it was presented on the walls as well as the floor of the tank. We also had greater success with relatively lower moisture levels, minimal handling and tank cleaning, the provision of natural hides (palm fronds, which are the species’ preferred shelter site in the wild), and when keeping neonates and juveniles with adults rather than separating them. A key lesson from this process was the importance of allowing time and resources to identify the optimal husbandry conditions for each species. It took approximately twelve months to stabilise the population, and then another twelve months before we began to see consistent population growth. Finally, after additional husbandry changes that included the provision of food high up on the sides of the tank and a reduction in tank disturbance, we began to see significant population growth. Our experience suggests that individual snail species’ requirements are likely to be highly specific, and few generalisations can be made so far on how to establish breeding programmes for ’at risk’ snail species. However, reducing causes of stress for the snails appears to have been one of the most significant factors in increased adult survival.

At the outset of the program we identified four measures of success: (1) consistency of breeding, with births occurring in every tank; (2) consistent survivorship of young, with a high percentage (at least 60%) of young reaching adulthood; (3) consistent longevity, with 70% of adults reaching a common age (and with aims of determining age at adulthood and lifespan); and (4) overall increase of project populations.

Two of these milestones have been met (consistency of breeding and population growth). Preliminary results indicate consistent longevity, but this needs to be confirmed once we establish a more effective method of marking individual snails. The fourth measure, survivorship of juveniles to adulthood, has not been reached, but the figures have improved during the duration of the study to 33% in March 2024. It may be that survivorship of neonates in the wild is lower than anticipated and that our initial target of 60% was unrealistically high. Over time, as data accumulate, we expect to get a more realistic estimate of survivorship for this species, at least in an intensely managed population. The improved survivorship of neonates and juveniles will inform management decisions that might bring us closer to another goal of the program: to reach a large enough population size to attempt releases of zoo-bred snails into the wild, if deemed an appropriate conservation management action.

At the outset of our study we knew that *A*. *campbellii campbellii* shared the trait of ovoviviparity with Pacific Island groups Partulidae and Achatinellinae, which have experienced extremely high rates of decline and extinction. However, where the latter groups measure their lifespan in years (for example, members of achatinellid genera *Achatinella* and *Partulina* reach maturity between four and seven years [4]), our study shows that *A*. *campbellii campbellii* reaches maturity after only 3–4 months and lives no more than a single year. There is also a difference in rate of reproduction, which is measured at four to seven offspring per year in *Achatinella* and *Partulina* [4], compared to approximately fortnightly birth intervals in *A*. *campbellii campbellii*. Despite its still low lifetime reproductive output, the faster growth and more rapid rate of reproduction may allow *A*. *campbellii campbellii* greater resilience in the face of ongoing predation. Moreover, we are fortunate in the absence of the carnivorous snail *Euglandina rosea*, which is thought to pose a greater threat to *Achatinella* spp. than rodent predation since it preys on all size classes, and may attack in repeated waves, leading to a higher likelihood of complete population extirpation [4].

The husbandry program has provided a great deal of valuable data on the growth, diet, longevity, survivorship and reproduction of *A*. *campbellii campbellii*. We have prioritized the minimization of disturbance and so have elected not to measure growth weekly or to place cameras in the enclosures to observe behaviour, but may consider the implementation of those measures in the future. Now that the breeding program for *A*. *campbellii campbellii* is well-established, it provides a starting point to add other threatened Norfolk Island snails to the breeding program, if deemed necessary. Other planned additions to the program include studies of the population genetics of both wild and captive populations.

#### Exclosures

We implemented preliminary trials of predator-proof exclosures *in-situ* to see if these could be useful conservation tools to protect breeding adults from predation. The intent of the exclosure trial was two-fold; first, to determine whether exclosures successfully excluded predators, retained adults, and had minimal impact on the snails; and second, to gather preliminary data on the required frequency of leaf litter replenishment. Regular provision of fresh leaf litter / logs for shelter and food is necessary in small exclosures [39], and determining the frequency of litter replenishment is a critical factor in their success and the eventual cost-effectiveness of their implementation. The frequency of replenishment depends on the food preferences of the snails. If they are primarily feeding directly on decaying vegetation, it should be possible to provide a large amount of leaf litter and replenish the vegetation at longer intervals (e.g. one to two months). However, biofilm feeders would need more regular replenishment of litter and may even fail to thrive in such a confined situation, (D. Sischo, pers. comm.). Our aim was not to determine exactly what food was being eaten by the snails, but instead, to determine whether exclosures were a successful tool for conservation and the appropriate frequency of leaf litter replenishment.

We established that our exclosures were successful in retaining the marked snails (with only one breach, soon rectified) and in keeping out predators, and that snails inside the exclosures were breeding. Some mortality of snails within the exclosures was observed. While this may simply be a result of the species’ short lifespan, it also raises the possibility that the experimental design impacted the snails. Prior to the experiment we identified replenishment of leaf litter as a critical factor in the success of the exclosures. A higher mortality rate was observed in phase 2, when leaf litter was replenished less frequently, suggesting inadequate food supply as a possible cause. However, factors such as exclosure placement (providing a less optimal microhabitat or lack of shelter sites) may also have a significant impact.

Our experimental design was hampered by the need to minimize the potential impact on these Critically Endangered snails, meaning that we have insufficient replication for statistical analysis and our results can only be considered as preliminary. However, our experiment suggests that our target taxa may be feeding on biofilm and therefore need weekly or fortnightly replenishment of food in their exclosures. This agrees with field observations of living snails but should be confirmed through further study. We recommend that in future a larger exclosure is used for the larger *A*. *campbellii campbellii*, to reduce the population density inside the exclosure and allow for a greater amount of litter to be added. We also recommend trials directly comparing weekly food replenishment with fortnightly and monthly replenishment, to determine the best protocol for this conservation intervention.

#### Habitat enhancement

Our primary measure of habitat enhancement was the addition of woody debris to the sites of *A*. *suteri* and *F*. *imitatrix*. It was difficult to measure the effect of this intervention; snails were observed to primarily use the bottom layer of logs, and a thorough search would result in dismantling and disturbing this environment. However, observations taken during regular surveys indicated snails were using the log-piles for shelter. This is a relatively low cost and low effort intervention that may be of considerable benefit in an environment where shelter sites are lacking.

Additional measures of habitat enhancement that could be trialed in the future include the addition of suitable debris to other habitat types, for example the provision of palm fronds in the habitat of *A*. *campbellii campbellii* or trialing a program of watering in very dry sites, possibly through the use of exclosures in order to measure success rates.

#### Predator control

The need for predator control was illustrated by the large numbers of *A*. *campbellii campbellii* shells showing signs of predation by rodents. Studies elsewhere have shown that rodent predation leaves characteristic bite marks by removing the middle whorls of the shell [50–52]. Shell damage corresponding with these bite marks has been observed in *A*. *campbelli campbelli*. Second, large numbers of chickens were observed at the sites where the first two species are found. There is only anecdotal evidence to suggest that chickens are a predator of endemic snails on Norfolk Island (Varman, pers. comm.), but they are prevalent and have a high potential for significant impact, both as a predator and through disturbance to the leaf litter. Introduced flatworm *Caenoplana coerulea* is another possible predator [53], but as there is no known method of control, it is not addressed here.

Many chickens were culled but no rats detected at Hundred Acres Reserve (habitat of *A*. *suteri*); while in the main site of *A*. *campbellii campbellii* in the Norfolk Island National Park, more rodents were killed than chickens. This suggests that the predation pressure may be different for these two species in their current locations. However, it may also be a factor of the different pest control measures used in the two locations. For example, regular chicken control work conducted in the Norfolk Island National Park may have been sufficient to decrease the chicken population within the main *A*. *campbellii campbellii* site before the advent of this project. Nonetheless, increasing chicken control appeared to have a significant effect on the chicken population at these two sites, indicating that this is a successful management strategy. In the long-term, it may be sufficient to engage in more cost-effective monthly rather than weekly chicken control. This may also reduce the learned response that chickens exhibited of avoiding staff members undertaking the culling.

Rodent control is one of the primary management tools used within the Norfolk Island National Park and Hundred Acre Reserve. Since 2020 there has been a network of 60 bait stations located in Hundred Acre Reserve. They are spaced 40 m apart and are baited monthly. The bait types are also rotated regularly to reduce resistance. The Norfolk Island National Park has operated a large bait station network since 1993, but a new control regime consisting of rotational baiting and trapping commenced in April 2022. Regular monitoring carried out in the Norfolk Island National Park using networks of 300 chew cards and 36 thermal cameras shows that the new control program has been very effective in reducing rodent activity across the park (S2 Appendix). With such a large rodent baiting network constantly in operation, it is difficult to ascertain the impact of increased rodent control specifically around the *A*. *campbellii campbellii* and the *A*. *suteri* sites; however, any extra kill is likely to have a positive impact. Monitoring in the National Park indicates that peak rodent activity occurs during the winter months (S2 Appendix), so extra rodent controls deployed during this time are likely to be most effective in protecting snails.

We did not undertake any direct measures or observations of predation pressure by rodents or chickens. Therefore, the significance of predation pressure remains a question to be further investigated. However, assuming that both are significant predators, we recommend continuing to implement increased rodent baiting and monthly chicken culling around populations of *A*. *campbellii campbellii* and *A*. *suteri*.

Island-wide rodent eradication has been successfully carried out on numerous islands worldwide [54], including recently on Lord Howe Island in Australia [55], with recorded benefits for a large suite of species including land invertebrates [56]. The majority of rodent eradications have been completed on uninhabited islands, with Lord Howe Island’s eradication only the second to be attempted on an inhabited island [55]. Norfolk Island’s relatively large and widespread settlement area would present huge challenges, but if it was led and championed by Norfolk Islanders [57], there is no doubt that island-wide eradication of rodents would be the optimal course of action for the island’s native fauna.

#### Monitoring

Our regular monitoring of the *A*. *campbellii campbellii* main site was carried out in order to assess changes in population over time. The dataset represents two years of monitoring so far, which is a relatively short span of time in which to distinguish between short-term, stochastic changes and long-term trends. The preliminary results we present appear to be closely linked to seasonal rainfall patterns (Fig 8C), indicating that this may be the strongest environmental factor affecting the abundance of the snails. We do not yet have sufficient data to be able to disentangle other possible drivers of change in the populations.

The highest proportion of rodent-predated shells was found in the first survey; however, this is likely to be an artefact of the survey method, since predated shells were removed after each survey to avoid recounting, meaning that the largest accumulation would have been present in the first survey. Discounting this first measure, proportion of rodent-predated empty shells ranged from 3% to 21% (average 14%) across the study period.

This raises the question: do these data imply that rodents are the primary threat? As yet we do not have sufficient data to make this assumption. While the breakage pattern of a shell that has been rodent-predated is relatively distinctive, other predators may leave no distinguishing marks. For example, a chicken may eat the whole snail including the shell, leaving no trace of its passing, whereas a flatworm will leave behind an intact shell, in all appearance identical to a snail that has died of natural causes. In order to resolve the conundrum of predation pressure, it will be necessary to engage in direct observation of predation using methods such as camera traps.

By counting dead shells as well as living individuals, we can theoretically determine whether a drop in live specimen count is due to lower detectability (i.e. species hiding more deeply due to very dry conditions) or mortality. Our results showed three occasions where the live specimen count dropped concurrently with an increase in dead shell count, but also, some occasions where the dead shell count increased without a corresponding decrease in live snail count, indicating that other factors are also at play. It may be that our sampling needs to be at a finer scale. The shells are a cumulative record, so will continue to accumulate in the months between sampling, while the live snail count will fluctuate. Monthly sampling may be required to develop a clearer picture.

Regular monitoring of *A*. *campbellii campbellii* will be continued in order to track long-term changes in population size. We have also begun a similar program of long-term monitoring of *A*. *suteri* in Hundred Acres Reserve.

### Future directions

Over the past three years we have gained considerable knowledge of Norfolk Island’s threatened snails; however, there remains much to be done. For some species, distribution data are still scanty. For all, we lack detailed and specific knowledge of predation pressure, growth, recruitment and longevity in the wild, and genetic structure of populations. We intend to continue regular surveys, focusing on species newly assessed as Critically Endangered for which data are lacking (such as *F*. *imitatrix* and *A*. *quintalae*). We will maintain the *ex-situ* population of *A*. *campbellii campbellii* at Taronga Zoo and might add a population of *A*. *suteri* and/or *F*. *imitatrix*, if deemed necessary. We also intend to study the population genetics of all three *Advena* species, in order to investigate the possible effects of population decline and fragmentation and past bottlenecks on the genetic health and structure of their populations. We will also undertake studies of predation pressure.

## Supporting information

S1 Appendix Conservation assessments of Norfolk Island land snails. We present conservation assessments of all Norfolk Island land snails previously assessed as EN, CR or EX following IUCN Red List guidelines (IUCN, 2019).
(DOCX)

S2 Appendix Rodent control in the Norfolk Island National Park. (DOCX)
