# Supplementary material for: The challenge of preventing extinctions: Lessons from managing threatened land snails on Norfolk Island
Source: PLoS One. 2024 Dec 16;19(12):e0314300. doi: 10.1371/journal.pone.0314300 (PMC11649095; doi:10.1371/journal.pone.0314300)
Supplement: S1 Appendix — We present conservation assessments of all Norfolk Island land snails previously assessed as EN, CR or EX following IUCN Red List guidelines (IUCN, 2019). (DOCX) [file pone.0314300.s001.docx]

S1 APPENDIX: Conservation assessments of Norfolk Island land snails

**Methods**

We reviewed the conservation assessments of all species previously assessed as EN, CR or EX following IUCN Red List guidelines [1]. We used GeoCAT (available online at<http://geocat.kew.org/>) to calculate the extent of occurrence (EOO) and area of occupancy (AOO) for each species.

The extent of occurrence (EOO) of each species is the area contained within a convex hull polygon around all known occurrence records. The area of occupancy (AOO) is the area contained within 2x2 km squares around each occurrence record. Before calculation of EOO and AOO, records were first checked for taxonomic and spatial accuracy and subfossil records were excluded. Calculations were made based on both recent data (collected in or after 2000) and historic data (specimens collected before 2000).

The following assessments are based on a comprehensive systematic review of all samples kept in the Malacological collection of the Australian Museum as well as eight field surveys undertaken between 2020 and 2023, which have specifically been designed to comprehensively survey the land snail fauna of Norfolk and Phillip Islands and to check presumably suitable habitats for the presence of any of the Critically Endangered species.

In addition, we incorporated all information on species occurrences shared with us by local conservationists and National Park rangers.

**Species conservation assessments**

***Advena campbellii*** (Gray, 1834)

**Differential diagnosis.** Shell moderately large, conical, with raised spire and strong peripheral angulation.

**Taxonomic remarks.** Originally described based on shell characters only. Contains two subspecies distinguished by shell shape and size: *A. campbellii campbellii*, originally present on Phillip and Norfolk Islands, now restricted to Norfolk Island, and *A. campbellii nepeanensis*, originally restricted to Nepean Island, extinct (see below). *Advena charon* is treated as a junior synonym of *A. campbellii campbellii* based on shell shape analysis [2]. Most recent revision provided anatomical data and a phylogeny based on mitochondrial DNA, demonstrating that this species is distinct from extant sister taxa *A. suteri* and *A. grayi* [2].

**Habitat and ecology.** Historically recorded from a range of native habitat types across the whole Norfolk Island Group, the species currently occurs only in Moist Palm Valley Forest revealing a strong ecological association with palms.

**Distribution.** Historically widespread on Norfolk Island and present on Phillip Island and Nepean Island; subfossil shells known from Cemetery Bay and Emily Bay [3]. Holotype of *campbellii* from Phillip Island (coll. 1830) but few other historic and no recent records from this location. Currently found only within the National Park at three locations in close proximity to each other.

**Conservation assessment.** Because the nominate form is the only surviving subspecies, the conservation status of *A. campbellii* is the same as that of the nominate form as assessed below.

***Advena campbellii campbellii*** (Gray, 1834)

Fig A in S1 Appendix

*Helix campbellii* Gray, 1834: 65 (Holotype: NHMUK 1982239, Phillip Island, under damp wood); Pfeiffer, 1848: 35-36.

*Helix campbelli*: Reeve, 1852: pl. 82, sp. 438, Reeve, 1854: pl. 127, sp. 765.

*Rotula campbelli*: Sykes, 1900: 141.

*Trochomorpha (Nigritella) campbelli*: Pfeiffer, 1878: 80.

*Advena campbelli var. charon* Preston, 1913: 526; Iredale, 1945: 66, pl. 4, fig. 17.

*Advena campbellii*: Iredale, 1945: 65-66; Smith, 1992: 226.

*Advena charon*: Smith, 1992: 226.

*Advena campbellii campbellii*: Hyman, Caiza & Köhler, 2023: 422.

**Differential diagnosis.** Characterised by a moderately large, conical shell with a raised spire and a strong peripheral angulation; colour can be pale golden-brown above and chocolate brown below periphery or universally golden-brown.

**Taxonomic remarks.** Originally described based on shell characters only. *Advena charon* is treated as a junior synonym based on shell shape analysis [2].

**Habitat and ecology.** Currently known only from Moist Palm Valley Forest, living under fallen palm frond bases on the ground. Appears to have a strong association with palms. Historic distribution data suggests that it may have once been found in coastal forest as well.

**Distribution.** Historically widespread on Norfolk Island and present on Phillip Island; subfossil shells known from Cemetery Bay and Emily Bay [3]. Holotype from Phillip Island (coll. 1830) but few other historic and no recent records from this location. More recent records primarily from the Norfolk Island National Park; exceptions include three specimens from Cascade / Simons Water in 1972 (AM C.435581) and one specimen from Bumbora Reserve in 1999 (AM C.392735). Live subpopulations recorded from Mt Cross until mid-1980 and Palm Glen Track around 1991 (Varman, pers. comm.); since believed extirpated in these locations. Currently found only in three small gullies on the southern slopes of Mt Pitt, Norfolk Island National Park, and private properties immediately adjacent to the National Park. Of these three subpopulations, one is significantly larger than the other two; one of the smaller subpopulations was significantly diminished in November 2022, possibly due to heavy rain and flooding.

**Population.** Only three subpopulations are known to exist based on the surveys undertaken since 2020. Maximum number of observed live specimens in a single survey was 197. Targeted searches for new subpopulations (with a focus on moist palm valleys) have been unsuccessful to date. Historic AOO = 32.0 km^2^; historic EOO = 19.9 km^2^. Recent AOO = 12.0 km^2^, recent EOO = 0.4 km^2^.

**Generation time.** The generation time of *A. campbelli* is not known; however, data from snails in captivity suggests that this species reaches maturity at approximately 5 months and that its lifespan is 10-12 months [4].

**Conservation and threats.** Predation by introduced rodents is inferred to be a significant threat to this species since rodent-predated shells have been observed at all known locations. We infer that another possible threat is predation by feral chickens, which have significantly increased in abundance over the past 30 years (Varman, pers. comm.). Chickens may also negatively impact habitat structure when foraging for food as they disturb the leaf litter layer. Most of the species’ range is in a protected area, minimising any ongoing effects from habitat disturbance. Climate change is inferred to be an emerging threat impacting average temperatures and moisture levels and the frequency and severity of extreme weather events.

**Conservation assessment.** Prior to 2020, the most recent record of *A. campbellii* were five empty shells collected in 2002. So, little information is available for the previous 10 years or three generations. However, this lack of data is largely a reflection of limited survey efforts as no targeted land snail surveys were carried out between April 2002 and March 2020. Given the known presence of the identified threat through this period, and the documented decline of the species from the time of first records in 1888, we infer a continued decline in area of occupancy, number of subpopulations and number of mature individuals through this period.

We assess *A. campbellii campbellii* as CRITICALLY ENDANGERED under Criterion B. It meets B1 (extent of occurrence < 100 km^2^) and B2 (area of occupancy < 10 km^2^) and meets conditions (a) (number of locations = 1) and (b) (continuing decline inferred in area of occupancy, number of subpopulations and number of mature individuals).

***Advena grayi*** (Sykes, 1900)

Fig A in S1 Appendix

*Fretum grayi* Sykes, 1900: 141, pl. 13, figs 6-7 (Syntypes: NHMUK 59.1.14.51, Norfolk Island, found dead, 2 specimens).

*Belloconcha elevata* Preston, 1913: 529 (Holotype: NHMUK 1915.1.4.1290, Nepean Is., off Norfolk Is; Paratypes: AM C.115681, Nepean Island, R. Bell, 1910-1912, 3 specimens); Iredale, 1945: 67, pl. 5, figs 13-15.

*Mathewsoconcha grayi*: Iredale, 1945: 67.

*Mathewsoconcha elevata*: Smith, 1992: 237.

*Advena grayi*: Hyman, Caiza & Köhler, 2023: 429.

**Differential diagnosis.** Characterised by a medium-sized to large conical shell with a raised spire, rounded periphery and closed umbilicus. Shell colour orange-brown, sometimes with a white peripheral band. Body grey-brown with reddish pigment (especially around the head).

**Taxonomic remarks.** Described based on shell alone. Recent taxonomic revision treated *Belloconcha elevata* as a junior synonym of *A. grayi* based on shell shape data, and genus *Mathewsoconcha* as a junior synonym of genus *Advena* based on morpho-anatomy and mitochondrial DNA [2]. *Advena grayi* is morphologically and genetically distinct from congeners *A. campbellii* and *A. suteri* [2].

**Habitat and ecology.** Living in New Zealand flax (*Phormium tenax*) on exposed hillside.

**Distribution.** Historically present on Norfolk, Phillip and Nepean Islands; Norfolk and Nepean Island specimens consist only of subfossils. Common in subfossil deposits in Cemetery Bay dating to 200-1,200 years ago [3]. Live-collected specimens were found on Jacky Jacky Ridge on Phillip Island in 1982. Targeted surveys in October 2020 recovered worn shells of indeterminate age from two locations. Targeted surveys in October 2022 revealed *M. grayi* living in New Zealand flax plants below Jacky Jacky Ridge.

**Population.** Global population size unknown, but recent observations indicate at least several hundred living specimens on Phillip Island (Tweed, pers. comm.). Historic AOO and EOO cannot be calculated due to lack of accurate locality data. Recent AOO = 8.0 km^2^; recent EOO = 0.2 km^2^.

**Generation time.** The generation time of *A. grayi* is not known. Congener *A. campbellii* reaches maturity at approximately 5 months and its lifespan is 10-12 months [4].

**Conservation and threats.** *Advena grayi* appears to be extirpated on Norfolk Island where it is recorded only as a subfossil, despite being common 200-1,200 years ago; however, it has survived on rodent-free Phillip Island. This indicates that rodent predation is likely to have played a considerable role in its decline. It is also likely that land clearing has historically been a key threatening process.

**Conservation assessment.** We assess *A. grayi* as VULNERABLE under Criterion D2 (typically: AOO < 20 km² or number of locations ≤ 5). We infer that there was a significant historic decline in the range of this species over 200 years ago, although historic AOO and EOO could not be calculated due to a lack of accurate locality data. Because we were unable to record any traces of snails in disturbed habitats, such as erosion scars and weed-dominated vegetation, we infer that the population is restricted to flax stands. In the medium term, we infer that there is no serious conservation threat to this habitat. However, climate change or progression of weeds may pose a threat to the species’ preferred habitat in the longer term. The population would also be highly vulnerable if rats or another introduced predator arrived on the island, or if there was significant habitat disturbance due to disease or an extreme weather event. Ongoing monitoring is advised to detect any change in distribution as well as population trends.

***Advena phillipii*** (Gray, 1834)

*Helix phillipii* Gray, 1834: 65; Pfeiffer, 1848: 192 (Syntypes: NHMUK 1982238, Phillip Island, 2 specimens).

*Helix phillipi*: Reeve, 1852: pl. 82, sp. 438.

*Fretum phillipii*: Sykes, 1900: 140.

*Fretum microstriatum* Preston, 1913: 526 (Holotype: NHMUK 1915.1.4.1286, Limestone Quarry, Norfolk Island; Paratypes: AM C.37182, Limestone Quarry, Norfolk Island, R. Bell, 1910-1912, 4 specimens).

*Mathewsoconcha microstriata*: Iredale, 1945: 66-67, pl. 5, figs 10-12; Smith, 1992: 237.

*Mathewsoconcha phillipii*: Iredale, 1945: 67; Smith, 1992: 237.

*Advena phillipii*: Hyman, Caiza & Köhler, 2023: 430.

**Differential diagnosis.** Characterised by a large subglobose shell with a slightly raised spire, rounded periphery and closed umbilicus. Colour pale yellow with white peripheral band.

**Taxonomic remarks.** Described based on shell alone. Recent taxonomic revision treated *Fretum microstriatum* as a junior synonym of *A. phillipii* based on shell shape data, and genus *Mathewsoconcha* as a junior synonym of genus *Advena* based on morpho-anatomy and mitochondrial DNA [2]. No anatomical or genetic data are available for *A. phillipii*.

**Habitat and ecology.** Unknown.

**Distribution.** Historically known from Norfolk Island and Phillip Island. Common in subfossil deposits in Cemetery Bay (200-1,200 years old) and Emily Bay (1,200-22,000 years old) [3]. Two syntypes (collected 1830, Phillip Island) represent the only non-fossil material in Museum collections. Two shells collected on Phillip Island in 1997 and identified by an expert as *M. phillippii* are now held in a private collection and could not be examined. Targeted surveys on Phillip Island in 2020 and 2022 were unsuccessful.

**Population.** Global population size unknown; however, in the absence of any verifiable, live or freshly dead specimens collected since 1830, it appears likely that this species is extinct.

**Conservation and threats.** It appears likely that this species was negatively impacted by predation from rodents, driving the Norfolk Island population to extinction. It appears to have persisted on rodent-free Phillip Island longer than on Norfolk, but was probably impacted by the extreme destruction of vegetation caused by feral animals on Phillip Island. The larger size of *A. phillipii* compared to *A. grayi* may have made it more difficult for it to survive in small patches of suitable vegetation.

**Conservation assessment.** EXTINCT. In the absence of any taxonomically verified, non-fossil specimens since 1830 (193 years), we recommend an IUCN species listing of Extinct.

***Advena suteri*** (Sykes, 1900)

Fig A in S1 Appendix

*Fretum suteri* Sykes, 1900: 140, pl 13, figs 10-11, 19 (Holotype: NHMUK 1914.1.7.455.45, Norfolk Island; Paratypes: NHMUK 1914.1.7.456–7, Norfolk Island, 2 specimens).

*Mathewsoconcha albuminata* Preston, 1913: 528 (Holotype: NHMUK 1915.1.4. 1289, near limestone quarry on SE coast of Norfolk Is; Paratypes: AM C.37153, nr limestone quarry, Norfolk Island, R. Bell, 1910-1912, 2 specimens); Iredale, 1945: 66, pl. 5, figs 7-9; Smith, 1992: 236.

*Mathewsoconcha belli* Preston, 1913: 528 (Holotype: NHMUK 1951.1.4.1301, Mt Pitt, Norfolk Is; Paratypes: AM C.38924, Mt Pitt, Norfolk Island, R. Bell, 1910-1912, 2 specimens); Iredale, 1945: 66; Smith, 1992: 236.

*Belloconcha compacta* Preston, 1913: 530 (Holotype: NHMUK 1915.1.4.1323, Nepean Is., off Norfolk Is; Paratypes: AM C.115680, Nepean Island, R. Bell, 1910-1912, 3 specimens; AM C.37177, Nepean Island, R. Bell, 1910-1912, 3 specimens); Iredale, 1945: 67, pl. 5, figs 19-21.

*Belloconcha norfolkensis* Preston, 1913: 530 (Syntypes: AM C.38926, Burnt Pine Valley, Norfolk Island, R. Bell, 1910-1912, 2 specimens).

*Mathewsoconcha vexillum* Preston, 1913: 529 (Holotype: NHMUK 1915.1.4.1282, limestone quarry, Norfolk Is).

*Mathewsoconcha suteri*: Iredale, 1945: 67; Smith, 1992: 237.

*Mathewsoconcha compacta*: Smith, 1992: 236-237.

*Advena suteri*: Hyman, Caiza & Köhler, 2023: 424.

**Differential diagnosis.** Characterised by a medium-sized, subglobose shell, with a slightly raised spire and apex and a rounded whorl profile. Shell colour orange-brown with a narrow white peripheral stripe; body colour grey-brown with a cream sole.

**Taxonomic remarks.** Described based on shell alone. Listed by the IUCN as *Mathewsoconcha belli* and by the EPBC Act as *Mathewsoconcha suteri*; however, recent taxonomic revision synonymised *Mathewsoconcha* with *Advena* based on mitochondrial DNA, and demonstrated *Mathewsoconcha belli* to be a junior synonym of *A. suteri* based on shell measurement data [2]. Other junior synonyms include *Mathewsoconcha albocincta*, *M. vexillum*, *M. compacta*, *Belloconcha compacta* and *B. norfolkensis*. Reproductive morphology and mitochondrial DNA indicate that this species is distinct from *A. campbellii* and *A. grayi* [2].

**Habitat and ecology.** Found in Coastal Pine and White Oak Forest, living under and inside rotting logs on the ground.

**Distribution.** Historically present on both Norfolk and Nepean Islands; common in subfossil deposits from Emily Bay and Cemetery Bay, the latter dating to 200-1,200 years old [3, 5]. Fresh shells recorded from few localities historically, including Mt Pitt, Mt Cross, Ball Bay, the Cascades and 100 Acre Reserve. In recent years, single fresh shells collected in Norfolk Island National Park in 1981 and 2002. Currently known only from Hundred Acre Reserve.

**Population.** A single living subpopulation is currently known. The maximum number of observed live specimens in a single survey is 165. Targeted searches of other remnant patches of Coastal Pine and White Oak Forest and last known records in the Norfolk Island National Park have been unsuccessful. Historic AOO = 28.0 km^2^, historic EOO = 14.4 km^2^. Recent AOO = 8.0 km^2^, EOO = 0.44 km^2^.

**Conservation and threats.** Land clearing and degradation has undoubtedly played a part in the contraction of the historic range of this species. Recent surveys have shown no signs of rodent-predated shells, but large numbers of feral chickens have been observed in the snails’ habitat. Another potential threat is climate change. Initial surveys in March 2020 (directly after Norfolk Island’s driest summer on record) showed hundreds of freshly dead shells and only two living specimens. Hundred Acre Reserve is a relatively open forest; increasing frequency and severity of spells of dry weather may render the microclimate too dry for the snails. To mitigate this threat and add shelter sites, piles of Norfolk Island pine logs have been added to the habitat.

**Conservation assessment.** Prior to observations made by the authors in the period 2020-2024, the most recently collected specimens of *A. suteri* were 15 specimens collected at two locations in 2002, so there is very little information available for the previous 10 years or three generations. However, this is largely a reflection of the limited survey effort in this period, with no targeted land snail surveys carried out between April 2002 and March 2020. The collections in 2002 represented two separate locations (one in the National Park and the other in Hundred Acre Reserve), whereas only a single location (Hundred Acre Reserve) is now known. This represents a continued decline in the number of subpopulations. We also infer a continued decline in area of occupancy, number of subpopulations and number of mature individuals based on the continued presence of the primary threats and a documented history of decline since first records were collected. There has also been extreme fluctuation in number of mature individuals observed during the period 2020-2024, with only two individuals detected in March 2020 compared to 165 individuals detected in November 2022.

We assess *A. suteri* as CRITICALLY ENDANGERED under Criterion B. It meets B1 (extent of occurrence < 100 km^2^) and B2 (area of occupancy < 10 km^2^) and meets conditions (a) (number of locations = 1), (b) (observed decline in number of locations; continuing decline inferred in area of occupancy, number of subpopulations and number of mature individuals) and (c) (extreme fluctuation in the number of mature individuals).

***Advena stoddartii*** (Gray, 1834)

*Caracolla stoddartii* Gray, 1834: 65

*Advena stoddartiii*: Hyman, Caiza & Köhler, 2023: 431.

**Differential diagnosis.** Characterised by a medium-sized, conical shell with a strongly angulate periphery and closed umbilicus. Shell colour chestnut and cream with variable patterns of banding and flammulations.

**Taxonomic remarks.** Described based on shell alone. Listed by the IUCN and the EPBC Act as *Quintalia stoddartii*; Quintalia is now considered a junior synonym of *Advena* based on shell morphology [2]. Contains three subspecies, also based on shell morphology: *A. stoddartii stoddartii* (restricted to Phillip Island), *A. stoddartii flosculus* (restricted to Norfolk Island) and *A. stoddartii intermedia* (restricted to Nepean Island and only known as a subfossil).

**Habitat and ecology.** Unknown. Apparently once widespread on Norfolk Island, inhabiting a range of habitat types including coastal and montane forest, as well as Phillip and Nepean Islands.

**Distribution.** *Advena stoddartii stoddartii*: known only from Phillip Island; represented in Museum collections only by syntypes collected in 1830. A single additional shell collected on Phillip Island in 1997 and identified by an expert as *A. stoddartii* is now held in a private collection and could not be examined. Targeted searches on Phillip Island by IH and FK in 2020 and 2022 were unsuccessful.

*Advena stoddartii flosculus:* historically known only from Norfolk Island; rare in Cemetery Bay fossil deposits and absent from Emily Bay deposits [3]; all Australian Museum specimens are Recent shells primarily collected prior to 1912 without locality data. Exceptions are singletons from Mt Pitt, Duncombe Bay and Ball Bay, the former collected around 1910 and the latter two collected pre-1945. More recently, twenty-seven specimens were reportedly collected in 1996-1997 in Norfolk Island National Park [5] but are not lodged in any museum collections; their identity could not be verified.

*Advena stoddartii intermedia:* Known only from Nepean Island subfossils; extinct. Habitat unknown.

**Population.** Global population size unknown; however, in the absence of any verifiable, live or freshly dead specimens collected since 1830, it appears likely that this species is extinct.

**Conservation and threats.** Rodent predation is likely to have been the most significant threat to this species, particularly given that it is one of the larger species in the Norfolk Island group.

**Conservation assessment.** EXTINCT. In the absence of any taxonomically verified, non-fossil specimens since pre-1945 (at least 78 years), we recommend an IUCN species listing of Extinct.

***Allenoconcha quintalae*** (Cox, 1870)

Fig B in S1 Appendix

*Helix quintalae* Cox, 1870: 82 (Syntypes: AM C.115346, Norfolk Island, in pine forests, in damp places under leaves, J. Brazier, June 1865, 2 specimens).

*Nanina (Thalassia) quintalae*: Pfeiffer, 1878.

*Charopa (Thalassia) quintalae*: Tryon, 1886: 212.

*Charopa quintalae*: Sykes, 1900: 143, fig. 2.

*Nancibella quintalae*: Iredale, 1945: 62; Smith, 1992: 238.

Allenoconcha quintalae: Hyman, Caiza & Köhler, 2023: 408.

**Differential diagnosis.** Characterised by its high adult whorl count of around 7 whorls and its very small, depressedly trochoidal shell with an angulate periphery.

**Taxonomic remarks.** Described based on shell alone. Placed in monotypic genus *Nancibella* by Iredale [6] and listed by the IUCN as *Nancibella quintalia*. A recent revision based on reproductive anatomy and mitochondrial DNA demonstrated a close relationship between this species and members of the genus *Allenoconcha*, and treated *Nancibella* as a junior synonym of *Allenoconcha* accordingly [2].

**Habitat and ecology.** Moist palm valley forest, found in leaf litter.

**Distribution.** Historically rare: known from only five shells in the AM collections (all collected before 1900, no precise location data); only a single shell known from fossil deposits at Emily Bay and Cemetery Bay, dating to between 1,200 and 22,000 years old. Iredale [6] noted a single specimen found in litter from lower slopes of Mt Pitt. No fresh material observed 1983-1990 despite regular collecting, leading to listing as Extinct on the IUCN Red List of Threatened Species [7]. However, in later years shells observed from few locations in National Park (Varman pers. comm.). Neuweger et al. [5] reported collecting 21 specimens from large-scale litter collecting in rainforest on Mt Pitt; however, specimens cannot be located so identity is unconfirmed. Single live specimens collected by the authors on two occasions from Mt Cross / Mt Bates regions in Norfolk Island National Park (2020, 2022).

**Population.** Global population size unknown. Based on the scarcity of historical samples, this species appears to be rare and in low abundance. Lack of historical locality information makes it difficult to assess changes in distribution and it is not possible to calculate an historic AOO or EOO. Recent AOO: 0.0 km^2^; recent EOO: 4.0 km^2^.

**Conservation and threats.** Current known distribution is completely contained within a protected area. Rodent predation may pose a threat.

**Conservation assessment.** There is very little information available for the previous 10 years or three generations. Only a single subpopulation is known, and based on finding only two specimens during targeted land snail surveys in the period 2020-2024, we infer a very small population size of <50 specimens. Based on the (unverified) collection of 21 specimens in 1999 [5], we infer a continued decline in area of occupancy and number of mature individuals.

We assess *A. quintalae* as CRITICALLY ENDANGERED under Criteria B and D. It meets B1 (extent of occurrence < 100 km^2^) and B2 (area of occupancy < 10 km^2^) and meets conditions (a) (number of locations = 1) and (b) (continuing decline inferred in area of occupancy and number of mature individuals). It also meets criterion D1 (inferred population size of fewer than 50 mature individuals).

***Fanulena amiculus*** (Iredale, 1945)

Fig B in S1 Appendix

*Dolapex amiculus* Iredale, 1945: 64 (Holotype: AM C.115348, Mt Pitt, Norfolk Island, H.C. Quintal, 1945; Paratype: AM C.115349, Mt Pitt, Norfolk Island, J. Brazier, Jun 1865, 1 specimen); Smith, 1992: 228.

*Dolapex fraternu*s Iredale, 1945: 64 (Holotype: AM C.115347, Mt Pitt, Norfolk Island, J. Brazier, Jun 1865); Smith, 1992: 228.

*Fanulena amiculus* Hyman, Caiza & Köhler, 2023: 415.

**Differential diagnosis.** Characterised by its moderately high spire, adult whorl count of around 6 whorls, predominantly spiral protoconch sculpture, the presence of periostracal blades on the teleoconch ribs, and only a small columellar tooth. It is paler in colour than *F. insculpta* and has more prominent flammulations. Occasionally a specimen with a white shell is seen.

**Taxonomic remarks.** Described based on shell alone and initially placed in monotypic genus *Dolapex* (Iredale, 1945). *Dolapex* was treated as a junior synonym of *Fanulena* in a recent taxonomic revision based on reproductive morphology and mitochondrial DNA [2].

**Habitat and ecology.** Ground-dwelling, living under logs and in leaf litter. Found in Moist Palm Valley forest, Moist Upland Hardwood Forest, and Coastal Pine and White Oak Forest.

**Distribution.** Historically recorded from Mt Pitt, Duncombe Bay, Ball Bay and around Kingston. Subfossil specimens likely belonging to this species present in Emily Bay deposits (1,200-22,000 years old) [3], albeit with higher whorl counts (6-7 whorls) than living subpopulations. Live subpopulations currently known from Norfolk Island National Park (Duncombe Bay, end of Marsh Rd) and 100 Acre Reserve (last collected 2002).

**Population.** Global population size unknown. The species appears to have a patchy distribution but to be locally common in some areas. Historic AOO = 24.0 km^2^, historic EOO = 8.9 km^2^. Recent AOO = 16.0 km^2^, EOO = 4.2 km^2^.

**Conservation and threats.** The current distribution of this species is within protected areas. Predation by rodents and chickens could be a present threat, but there is no clear evidence to reflect this for smaller species (e.g. presence of rodent-predated shells).

**Conservation assessment.** While recent EOO and AOO meet the criterion for a listing of EN based on Criterion B, there are no signs of ongoing population decline. Given that the AOO is under 20 km^2^ and there is a small number of subpopulations, we recommend a listing of VULNERABLE under criterion D2.

***Fanulena imitatrix*** (Sykes, 1900)

Fig B in S1 Appendix

*Medyla imitatrix* Sykes, 1900: 142, pl. 13, figs 1-2 (Holotype: NHMUK 59.1.14.58, Norfolk Island, under dead leaves and wood).

*Lutilodix imitatrix*: Iredale, 1945: 63; Smith, 1992: 236.

*Fanulena imitatrix*: Hyman, Caiza & Köhler, 2023: 417.

**Differential diagnosis.** Characterised by a radially sculptured protoconch, high spire, an adult whorl count of 6.0-6.5 whorls, and the presence of periostracal hairs on the teleoconch [2].

**Taxonomic remarks.** Described based on shell alone. Listed by the IUCN as *Lutilodix imitatrix*. Recent taxonomic revision based on mitochondrial DNA and reproductive morphology demonstrated a close relationship with *Fanulena* and treated *Lutilodix* as a junior synonym of *Fanulena* accordingly.

**Habitat and ecology.** Coastal Pine and White Oak Forest, living under logs.

**Distribution.** Historical distribution poorly understood; original description lacked locality data. Common in subfossil deposits at Emily Bay and Cemetery Bay [3, 5]. Recorded by Varman [3] as uncommon live; Neuweger et al. [5] recorded a single specimen from Mt Pitt but identity could not be confirmed. Currently only known from the central part of Selwyn Reserve.

**Population.** Global population size unknown. Recent live specimen counts of the only known subpopulation have never exceeded 25 specimens, but given the small body size, detectability is likely to be very low. Based on the scarcity of historical samples, this species appears to be rare and in low abundance. There are insufficient samples to calculate historic AOO and EOO. Recent AOO: 4.0 km^2^; recent EOO: 0.001 km^2^.

**Conservation and threats.** The most significant threat to this species historically is likely to have been loss of habitat. It is currently found in one small region of Selwyn Reserve, which is a recreational reserve and not a protected area. Management actions have been taken to reduce foot traffic through this area and to add woody debris suitable for shelter sites. Rodent predation is likely to be a present threat; currently no rodent control is undertaken in Selwyn Reserve.

**Conservation assessment.** While subfossil deposits at Emily Bay and Cemetery Bay show that *F. imitatrix* was once more widespread across the island, there is very little information available for the previous 10 years or three generations. Only a single subpopulation is known. We infer a continued decline in area of occupancy and number of mature individuals based on the continued presence of rodent predation.

We assess *Fanulena imitatrix* as CRITICALLY ENDANGERED under Criterion B. It meets B1 (extent of occurrence < 100 km^2^) and B2 (area of occupancy < 10 km^2^) and meets conditions (a) (number of locations = 1) and (b) (continuing decline inferred in area of occupancy and number of mature individuals).

***Fanulena perrugosa*** Iredale, 1945

*Fanulena (Parcolena) perrugosa* Iredale, 1945: 63 (Syntypes: AM C.40598, Norfolk Island, R. Bell, 1910-1912, 2 specimens).

*Fanulena perrugosa*: Smith, 1992: 230; Hyman, Caiza & Köhler, 2023: 417.

**Differential diagnosis.** Characterised by its small size, angulate rather than keeled periphery, strongly raised spire and high adult whorl count of around 7 whorls.

**Taxonomic remarks.** Species description is shell-based. Shell measurements show this species to be clearly distinct from all congeners [2].

**Habitat and ecology.** Unknown.

**Distribution.** Only represented in AM collections by four subfossil shells from two locations: two syntypes from an unknown location (coll. 1910-1912) and two shells from Emily Bay (coll. 1999). Never collected alive.

**Population.** No living individuals of this species were ever recorded. The small number of recorded subfossil shells suggests that this species was historically rare with low abundance.

**Conservation and threats.** No current threats are known.

**Conservation assessment.** EXTINCT. In the absence of any non-fossil specimens and the lack of evidence for a living population, we confirm the current IUCN species listing of Extinct.

**References**

1. IUCN [International Union for the Conservation of Nature]. Guidelines for using the IUCN Red List categories and criteria, Version 14. 2019. Accessed April 2021.Available from: <https://www.iucnredlist.org/resources/redlistguidelines>

2. Hyman IT, Caiza J, Köhler F. Systematic revision of the microcystid land snails endemic to Norfolk Island (Gastropoda: Stylommatophora) based on comparative morpho-anatomy and mitochondrial phylogenetics. Invertebr Syst. 2023;37(5–6), 334–443.<https://doi.org/10.1071/IS22049>

3. Varman RVJP. Conchological Survey 1983-90: Manuscript of Land Mollusca Fossiliferous and Present Day. Unpublished manuscript; 1991.

4. Daly A, Williams Clow T, Hyman IT, Bonson P, Finlayson B. Husbandry Manual for the Campbell’s Keeled Glass Snail (*Advena campbellii*, Gastropoda: Pulmonata: Microcystidae) and *Mathewsoconcha suteri* (Gastropoda: Pulmonata: Microcystidae). 2002. Available from:<https://aszk.org.au/wp-content/uploads/2023/01/NI-land-snails-husbandry-manual-November-22.pdf>

5. Neuweger D, White P, Ponder WF. Land snails from Norfolk Island sites. Rec Aust Mus Suppl. 2001;27, 115-122.

6. Iredale T. The land Mollusca of Norfolk Island. Aust Zool. 1945;11: 46-71.

7. Ponder WF, Varman R. *Nancibella quintalia*. The IUCN Red List of Threatened Species 1996; e.T14318A4431366. Available from:<https://dx.doi.org/10.2305/IUCN.UK.1996.RLTS.T14318A4431366.en>. Downloaded on 18 February 2021.


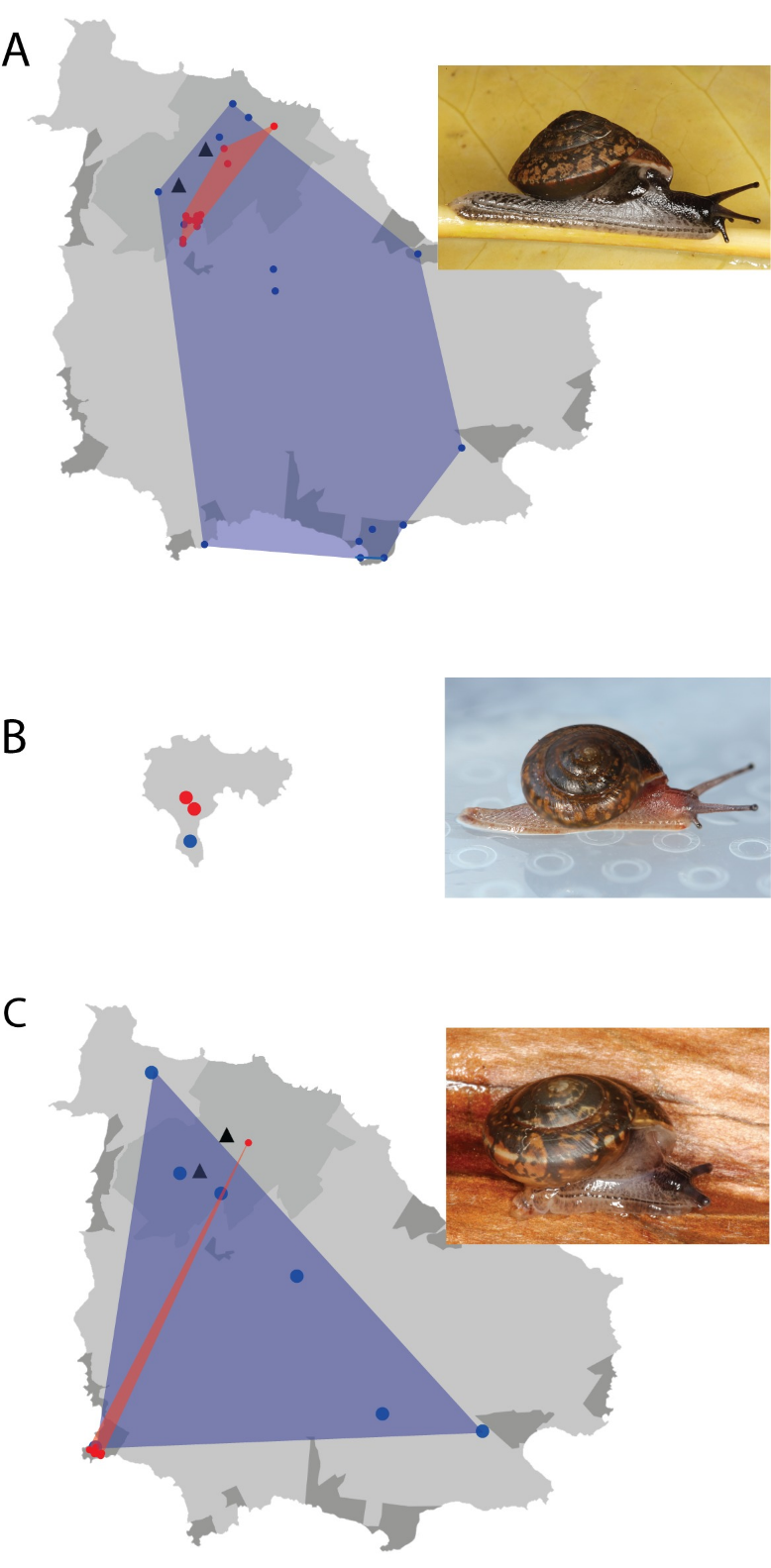


**Fig A in S1 Appendix. Distribution maps showing the Historic and Recent EOO for extant *Advena* species.**

A. *Advena campbellii campbellii*. B. *Advena grayi*. C. *Advena suteri*. The Norfolk Island National Park is shown in mid-grey and the reserves in darker grey. Recent records refer to live or freshly dead specimens collected from 2000 to the present. Historical records refer to live or freshly dead specimens collected before 2000.


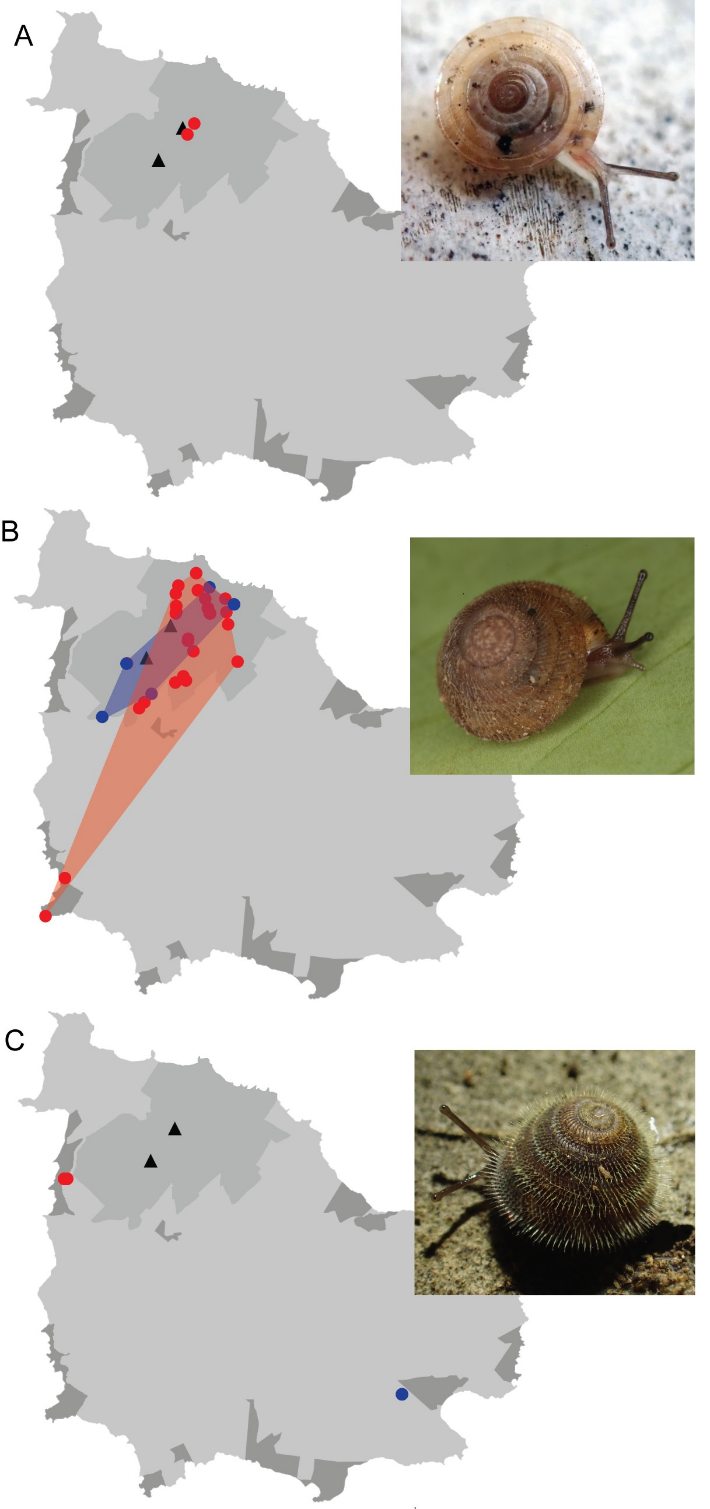


**Fig B in S1 Appendix. Distribution maps showing the Historic and Recent EOO for extant *Allenoconcha* and *Fanulena* species.**

A. *Allenoconcha quintalae*. B. *Fanulena amiculus*. C. *Fanulena imitatrix*. The Norfolk Island National Park is shown in mid-grey and the Reserves in darker grey. Recent records refer to live or freshly dead specimens collected from 2000 to the present. Historical records refer to live or freshly dead specimens collected before 2000.
