# Supplementary material for: The challenge of preventing extinctions: Lessons from managing threatened land snails on Norfolk Island
Source: PLoS One. 2024 Dec 16;19(12):e0314300. doi: 10.1371/journal.pone.0314300 (PMC11649095; doi:10.1371/journal.pone.0314300)
Supplement: S2 Appendix — (DOCX) [file pone.0314300.s002.docx]

S2 APPENDIX: Rodent control in the Norfolk Island National Park

As a result of extensive research, rodent control methods used in the Norfolk Island National Park changed in April 2022. At this time, a new control regime consisting of rotational baiting and trapping was begun. Baiting occurs in defined, intensively treated areas generally covering approximately 20% of the park at any one time, whilst mechanical traps are regularly deployed, moved and then re-deployed in identified “hotspot” locations. Subsequent monitoring using a network of 300 chew cards stratified by habitat type shows that the new control program has been very effective in reducing rodent activity across the park (Fig C). Monitoring with thermal cameras indicates that peak rodent activity occurs during the winter months, so extra rodent controls deployed during this time are likely to be most effective in protecting snails (Fig D).

**
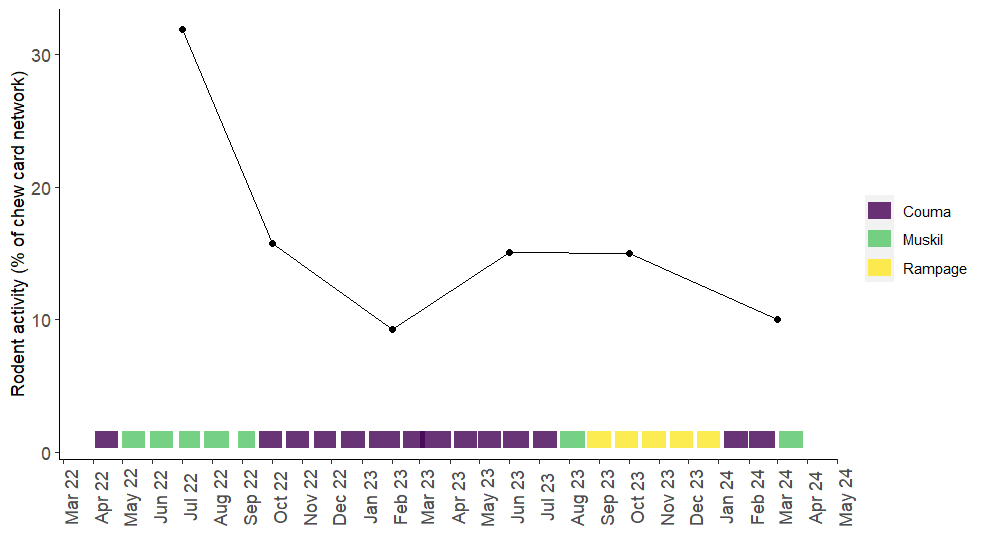
**

**Fig C.** **Rodent activity as a percentage of the chew card network (n=300), bait type and timing.**

**
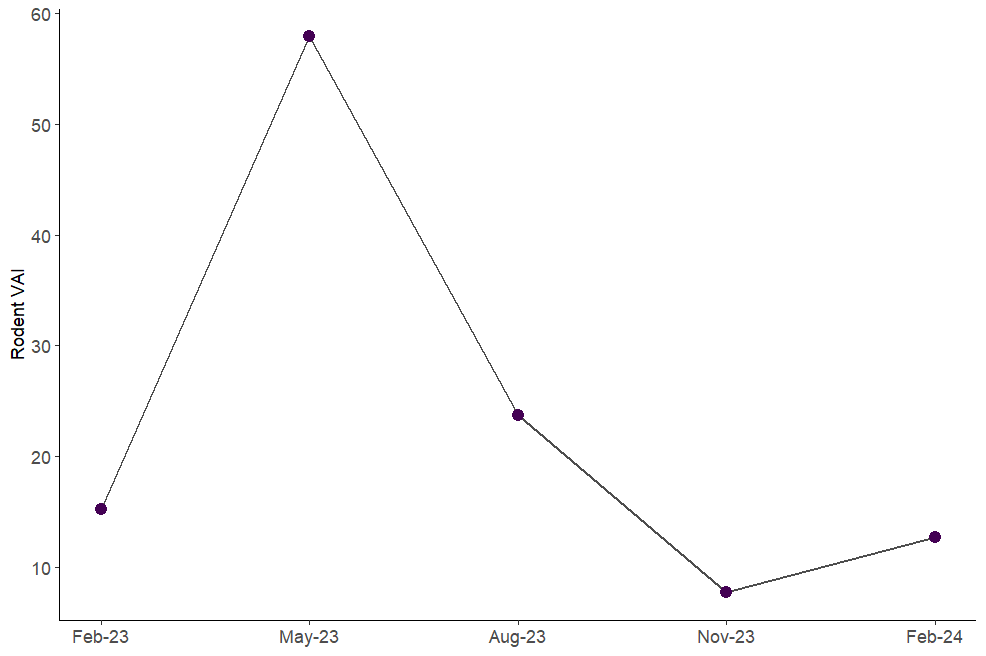
**

**Fig D:** **Rodent Visitation Abundance Index.**

Data is collected from thermal camera recordings from 36 sites. Cameras remain in-situ for a period of 1 week at each site. The VAI is a measure of the average number of visits by a rodent to a device, or “pressure” within the park.
